# P2Y_13_ receptor deficiency favors adipose tissue lipolysis and worsens insulin resistance and fatty liver disease

**DOI:** 10.1172/jci.insight.175623

**Published:** 2024-03-12

**Authors:** Thibaut Duparc, Emilia Gore, Guillaume Combes, Diane Beuzelin, Julie Pires Da Silva, Vanessa Bouguetoch, Marie-Adeline Marquès, Ana Velazquez, Nathalie Viguerie, Geneviève Tavernier, Peter Arner, Mikael Rydén, Dominique Langin, Nabil Sioufi, Mohamad Nasser, Cendrine Cabou, Souad Najib, Laurent O. Martinez

**Affiliations:** 1 LiMitAging, Institute of Metabolic and Cardiovascular Diseases (I2MC), University of Toulouse, INSERM, Université Toulouse III - Paul Sabatier (UPS), UMR1297, Toulouse, France.; 2 Institut Hospitalo-Universitaire HealthAge, (IHU HealthAge), INSERM, Toulouse University Hospital, Toulouse, France.; 3 Lifesearch SAS, Toulouse, France.; 4 MetaDiab, I2MC, University of Toulouse, INSERM, UPS, UMR1297, Toulouse, France.; 5 Department of Medicine, Karolinska Institutet, Karolinska University Hospital Huddinge, Stockholm, Sweden.; 6 Biochemistry Laboratory, Toulouse University Hospital, Toulouse, France.; 7 Institut Universitaire de France (IUF), Paris, France.

**Keywords:** Hepatology, Metabolism, Adipose tissue, G protein&ndash;coupled receptors, Glucose metabolism

## Abstract

Excessive lipolysis in white adipose tissue (WAT) leads to insulin resistance (IR) and ectopic fat accumulation in insulin-sensitive tissues. However, the impact of G_i_-coupled receptors in restraining adipocyte lipolysis through inhibition of cAMP production remained poorly elucidated. Given that the G_i_-coupled P2Y_13_ receptor (P2Y_13_-R) is a purinergic receptor expressed in WAT, we investigated its role in adipocyte lipolysis and its effect on IR and metabolic dysfunction-associated steatotic liver disease (MASLD). In humans, mRNA expression of P2Y_13_-R in WAT was negatively correlated to adipocyte lipolysis. In mice, adipocytes lacking P2Y_13_-R displayed higher intracellular cAMP levels, indicating impaired G_i_ signaling. Consistently, the absence of P2Y_13_-R was linked to increased lipolysis in adipocytes and WAT explants via hormone-sensitive lipase activation. Metabolic studies indicated that mice lacking P2Y_13_-R showed a greater susceptibility to diet-induced IR, systemic inflammation, and MASLD compared with their wild-type counterparts. Assays conducted on precision-cut liver slices exposed to WAT conditioned medium and on liver-specific P2Y_13_-R–knockdown mice suggested that P2Y_13_-R activity in WAT protects from hepatic steatosis, independently of liver P2Y_13_-R expression. In conclusion, our findings support the idea that targeting adipose P2Y_13_-R activity may represent a pharmacological strategy to prevent obesity-associated disorders, including type 2 diabetes and MASLD.

## Abstract 2

The P2Y13 receptor inhibits adipose tissue lipolysis, and loss of this receptor in mice promotes insulin resistance and fatty liver disease.

## Graphical abstract

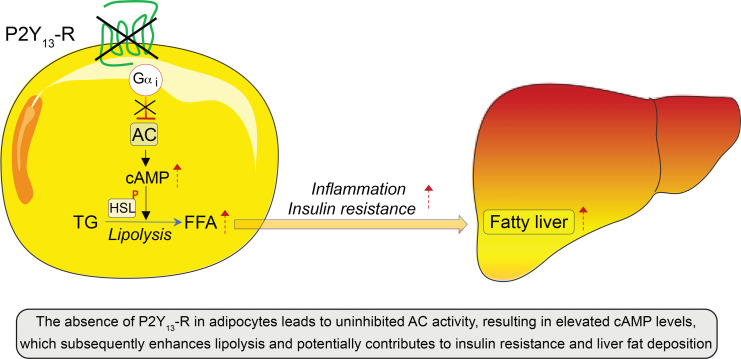

## Introduction

White adipose tissue (WAT) functions as a metabolic and endocrine organ and is composed of a specialist cell type known as adipocytes. The primary role of adipocytes is to store excess nutrient energy as triglycerides (TGs). During fasting or physical exercise, lipolysis in adipocytes breaks down TGs into free fatty acids (FFAs) and glycerol, providing energy substrates to other tissues. Lipolysis involves 2 major lipases: adipose triglyceride lipase (ATGL) and hormone-sensitive lipase (1). ATGL hydrolyzes TGs to diglycerides (DGs), while hormone-sensitive lipase converts DGs to monoglycerides (MGs), releasing FFAs at each step. The non-rate-limiting mono-acylglycerol lipase completes MG hydrolysis, releasing FFA and glycerol.

The major pathway leading to lipolysis is the activation of adenylate cyclase (AC) and subsequent cAMP production. The resulting increase in intracellular cAMP leads to protein kinase A–mediated (PKA-mediated) activation of hormone-sensitive lipase and subsequent release of FFA and glycerol. Lipolysis is mainly stimulated by catecholamines, which bind to G protein–coupled receptors (GPCRs) coupled to a Gα_s_ protein, thereby increasing cAMP production. Conversely, insulin is the most potent antilipolytic molecule, acting by reducing cAMP levels through phosphodiesterase activation (1). Additionally, compounds such as short-chain fatty acids, β-hydroxybutyrate, and lactate negatively regulate AC activity and inhibit lipolysis through G_i_-coupled receptors.

Dysfunctional white adipocyte lipolysis links to insulin resistance, obesity, and related disorders such as type 2 diabetes (T2D), dyslipidemia, and metabolic dysfunction-associated steatotic liver disease (MASLD, formerly NAFLD) (2–4). Elevated unstimulated lipolysis during obesity leads to increased FFA levels (2, 3), peripheral insulin resistance, lipotoxicity, and ectopic fat accumulation, particularly in the liver (5–7). Therefore, identifying drug targets to improve adipocyte metabolic function is crucial for preventing obesity-related metabolic disorders.

Purinergic receptors (P2Y-Rs) are GPCRs that are activated by nucleotides (8). Recent studies have suggested a link between purinergic signaling in adipocytes and the regulation of lipolysis (9–11). Eight P2Y-Rs exist in mammals, coupled with different Gα proteins (8, 12). Among them, P2Y_13_-R (also named GPR-86) is activated by ADP and primarily coupled to Gα_i_ protein (13). In humans, *P2RY13* mRNA is detected in various tissues and cell types, with the highest expression found in the spleen, placenta, different brain regions, hepatocytes, bone marrow, lung, and subcutaneous adipocytes (14–17). In mice, P2Y_13_-R activity has been linked to neuronal protection against oxidative stress and microglial function (18–21), mesenchymal stem cells’ differentiation into osteoblasts (22, 23), alarmins’ release from airway epithelial cells in response to aeroallergens or respiratory viruses (24), and high-density lipoprotein (HDL) endocytosis by hepatocytes (25, 26). However, the contribution of P2Y_13_-R in adipocyte lipolysis and obesity-related metabolic disorders remains underresearched.

To address this issue, we determined the impact of P2Y_13_-R deficiency in mice on adipose tissue lipolysis and whole-body lipid and glucose homeostasis. We show that the absence of P2Y_13_-R results in enhanced adipocyte lipolysis and aggravates diet-induced insulin resistance and hepatic steatosis and fibrosis. We also examined whether P2Y_13_-R’s antilipolytic function affects ectopic fat accumulation in the liver. Our findings suggest that targeting P2Y_13_-R could be a potentially effective approach in treating metabolic dysfunction associated with obesity.

## Results

### The activity of P2Y_13_-R in white adipocytes has a negative regulatory effect on lipolysis.

**Figure 1:**
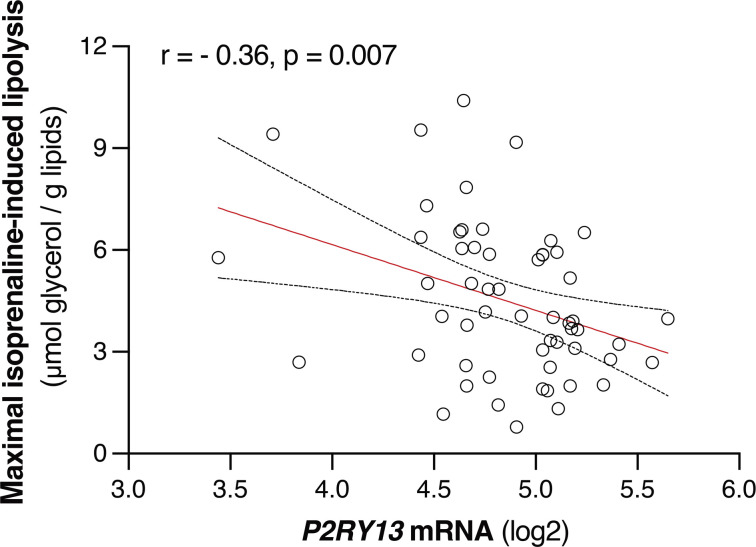
Relationships between *P2RY13* gene expression and lipolysis measures in human subcutaneous WAT samples. The scatterplot represents simple linear regression analysis, and the results are expressed as *r* (correlation coefficient) tested by Pearson correlation coefficient analysis (*n* = 56). WAT, white adipose tissue.

In humans, linear regression analysis showed a negative correlation between subcutaneous adipose tissue *P2RY13* mRNA expression and lipolytic activity in adipocytes from the same fat sample (*r* = –0.36, *P* = 0.007, Figure 1). This suggests that human P2Y_13_-R might play a role in regulating adipocyte lipolysis.

**Figure 2:**
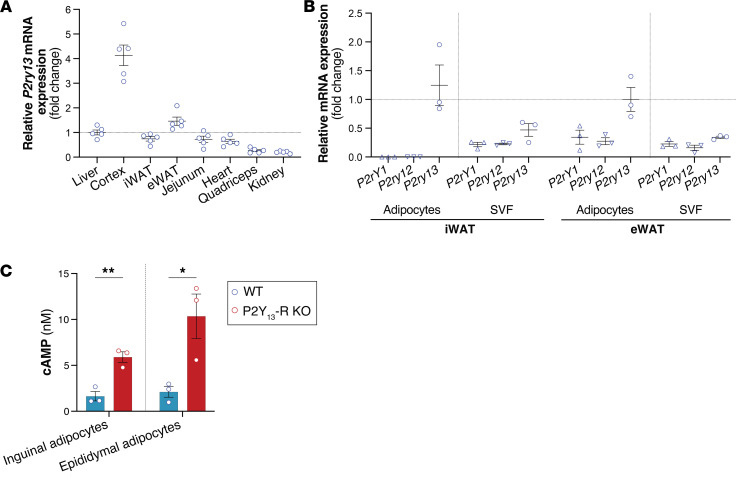
*P2ry13* gene expression and P2Y_13_-R activity in mouse primary adipocytes. (**A**) Relative mRNA expression levels of *P2ry13* in mouse tissue (*n* = 5 mice). (**B**) Relative mRNA expression levels of ADP purinergic receptors (*P2ry1*, *P2ry12*, and *P2ry13*) in primary adipocytes and SVF from iWAT and eWAT (*n* = 3 mice). (**C**) cAMP level in primary inguinal or epididymal adipocytes isolated from WT (control) and P2Y_13_-R–KO mice (*n* = 3 mice per group). Open blue and red circles represent WT and P2Y_13_-R–KO mice, respectively. mRNA expression data were normalized relative to the expression of *Rps29*. All data are expressed as mean ± SEM. *P < 0.05, **P < 0.01 (**C**, 2-tailed unpaired Student’s *t* test was used for genotype comparison). All results were obtained from 24-month-old mice fed a chow diet. eWAT, epididymal white adipose tissue; iWAT, inguinal white adipose tissue; KO, knockout; *Rps29*, ribosomal protein S29; SVF, stromal vascular fraction; WT, wild-type.

In mice, quantitative analysis of gene expression in major metabolically active tissues and organs revealed high relative mRNA expression of *P2ry13* in the cortex, followed by epididymal WAT (eWAT) and liver, and equally in inguinal WAT (iWAT; equivalent to subcutaneous WAT), jejunum, and heart, while being barely detectable in the quadriceps and kidney (Figure 2A). Similar results were obtained when considering the cycle threshold (Ct) values, except in the jejunum, where the expression of *P2ry13* was as low as in the quadriceps and the kidney (Supplemental Figure 1; supplemental material available online with this article; https://doi.org/10.1172/jci.insight.175623DS1). *P2ry13* expression was more than double in inguinal and epididymal adipocytes compared with the stromal vascular fraction (SVF), and the adipocyte expression of the 2 other genes coding for ADP receptors, *P2ry1* and *P2ry12*, was much lower than that of *P2ry13* (Figure 2B).

We next examined P2Y_13_-R activity in mouse primary adipocytes by measuring the level of intracellular cAMP. We found that cAMP production triggered by forskolin (FK), a direct AC activator, was significantly higher in both inguinal and epididymal adipocytes isolated from P2Y_13_-R–KO mice compared with those isolated from WT littermates (Figure 2C). This finding verifies that signaling through P2Y_13_-R in adipocytes involves coupling to G_i_ protein to inhibit AC.

**Figure 3:**
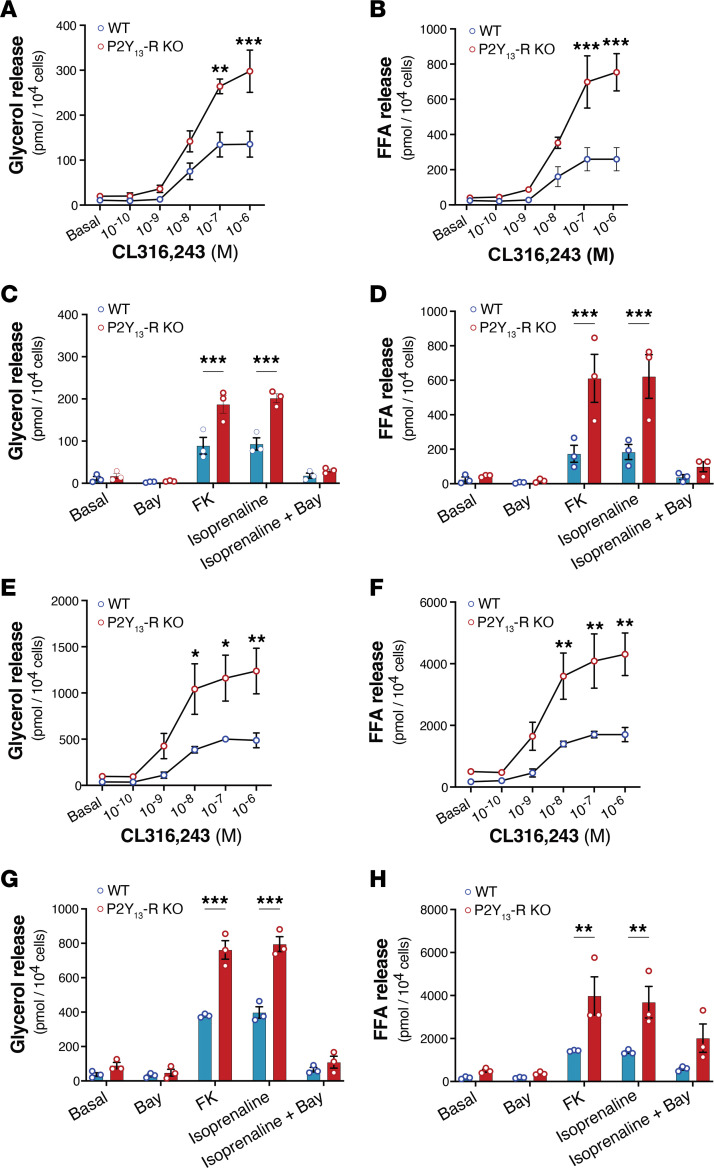
Lack of P2Y_13_-R in mouse primary adipocytes increases lipolysis. (**A**) Glycerol and (**B**) FFA release in culture media of adipocytes isolated from iWAT under basal condition or following stimulation with CL316,243 10^–10^ to 10^–6^ M (*n* = 3 mice per group). (**C**) Glycerol and (**D**) FFA release in culture me dia of adipocytes isolated from iWAT under basal condition or following stimulation with 10^–6^ M forskolin or 10^–6^ M isoprenaline (*n* = 3 mice per group). (**E**) Glycerol and (**F**) FFA release in culture media of adipocytes isolated from eWAT under basal condition or following stimulation with CL316,243 10^–10^ to 10^–6^ M (*n* = 3 mice per group). (**G**) Glycerol and (**H**) FFA release in culture media of adipocytes isolated from eWAT under basal condition or following stimulation with 10^–6^ M forskolin or 10^–6^ M isoprenaline (*n* = 3 mice per group). The experiments included a group treated with BAY 59-9435 (BAY), a potent and selective pharmacological inhibitor of hormone-sensitive lipase (*n* = 3 mice per group). Open blue and red circles represent WT and P2Y_13_-R–KO mice, respectively. All data are expressed as mean ± SEM. *P < 0.05, **P < 0.01, ***P < 0.001 (**A**–**H**, 2-way ANOVA followed by Bonferroni’s post hoc test was used for genotype comparison). All results were obtained from 24-month-old mice fed a chow diet. eWAT, epididymal white adipose tissue; FFA, free fatty acids; FK, forskolin; iWAT, inguinal white adipose tissue; KO, knockout; WT, wild-type.

In adipocytes, β-adrenergic receptors (β-ARs) trigger a lipolytic response mediated by the Gα_S_ protein, which leads to AC activation, cAMP production, and subsequent activation of hormone-sensitive lipase through PKA (2). To investigate whether P2Y_13_-R signaling affects lipolytic activity in primary adipocytes, we measured the extracellular release of lipolysis by-products, glycerol and FFA (Figure 3). The dose-dependent increase in lipolytic activity stimulated by CL316,243, a β_3_-AR agonist, was significantly higher in inguinal and epididymal adipocytes lacking P2Y_13_-R compared with those expressing the receptor (Figure 3, A and B, and Figure 3, E and F, respectively), consistent with the cAMP data. Similar results were observed when using FSK or isoprenaline, a nonselective β-AR agonist, to activate lipolysis (Figure 3, C and D, and Figure 3, G and H, respectively). Regardless of P2Y_13_-R expression, pharmacological inhibition of hormone-sensitive lipase with BAY suppressed isoprenaline-induced lipolysis. Collectively, the data indicate that P2Y_13_-R signaling in adipocytes inhibits cAMP production by AC. This tonic inhibition mediated by P2Y_13_-R results in reduced stimulation of lipolysis induced by β-ARs.

### The absence of P2Y_13_-R increases lipolysis and inflammation in adipose tissue.

**Figure 4:**
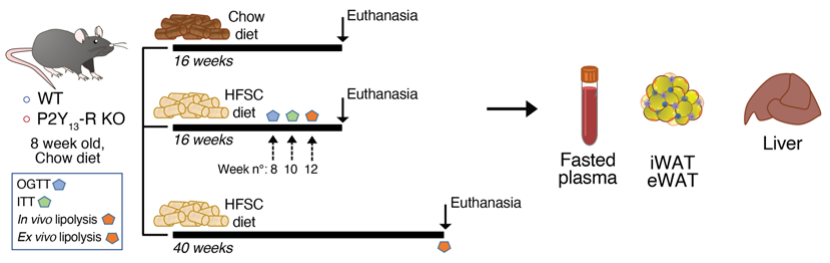
Experimental study design of metabolic studies in P2Y_13_-R–KO and WT (control) mice. eWAT, epididymal white adipose tissue; OGTT, oral glucose tolerance test; HFSC, high-fat high-sucrose high-cholesterol; ITT, insulin tolerance test; iWAT, inguinal white adipose tissue; KO, knockout; WT, wild-type.

To investigate the potential in vivo activity of P2Y_13_-R signaling in obesity-related metabolic disorders, we fed P2Y_13_-R–KO and control mice either a standard chow diet (CD) or a high-fat high-sucrose high-cholesterol (HFSC) diet for 16 weeks (27). An additional group was maintained on the HFSC diet for up to 40 weeks (Figure 4, study design).

Compared with the CD group, HFSC feeding resulted in increased total body weight, as well as increased iWAT, eWAT, and liver weight, with no significant differences observed between P2Y_13_-R–KO and control mice, except for body and liver weights, which were higher in P2Y_13_-R–KO mice after being on the HFSC diet for up to 40 weeks (Supplemental Figure 2, A–D).

**Figure 5:**
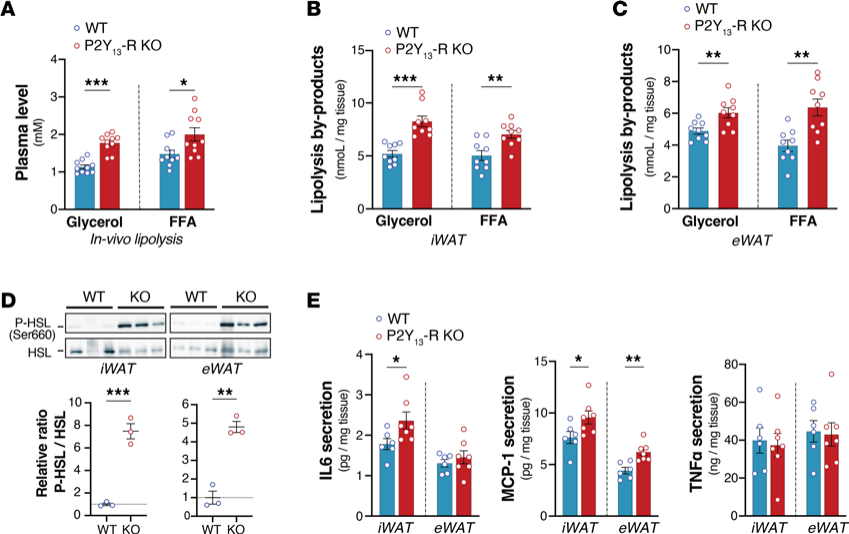
In vivo and ex vivo lipolytic activities of adipose tissues are increased in HFSC diet–fed P2Y_13_-R–KO mice. (**A**) Glycerol and FFA plasma levels from mice after being stimulated by CL316,243 (1 mg/kg body weight) for 15 minutes (*n* = 10 mice per group). (**B**) Glycerol and FFA release in supernatant of iWAT explants after being stimulated by 10^–6^ M CL316,243 (*n* = 9 mice per group). (**C**) Glycerol and FFA release in supernatant of eWAT explants after being stimulated by 10^–6^ M CL316,243 (*n* = 9 mice per group). (**D**) Western blot analysis and quantification of immunoblotting data of hormone-sensitive lipase phosphorylation at Ser660 (P-HSL) in iWAT and eWAT explants isolated from mice and stimulated with 10^–6^ M CL316,243 (*n* = 3 mice per group). (**E**) Concentrations of IL-6, MCP-1, and TNF-α in supernatant of iWAT and eWAT explants from HFSC diet–fed mice (*n* = 6 or 7 mice per group). Open blue and red circles represent WT and P2Y_13_-R–KO mice, respectively. All data are expressed as mean ± SEM. *P < 0.05, **P < 0.01, ***P < 0.001 (**A**–**E**, 2-tailed unpaired Student’s *t* test was used for genotype comparison). Results were obtained from mice fed HFSC for 12 weeks (**A**) or 40 weeks (**B**–**E**). eWAT, epididymal white adipose tissue; FFA, free fatty acids; HFSC, high-fat high-sucrose high-cholesterol; IL-6, interleukin-6; iWAT, inguinal white adipose tissue; KO, knockout; MCP-1, monocyte chemoattractant protein-1; TNF-α, tumor necrosis factor-α; WT, wild-type.

Lipolysis was assessed both in vivo at the 12-week HFSC feeding and ex vivo on adipose tissues explants at the end of the 40-week diet period. Consistent with the antilipolytic role of P2Y_13_-R signaling, in vivo β-AR–stimulated lipolysis, measured by plasma levels of glycerol and FFA after injection with CL316,243, was significantly increased in HFSC-fed mice lacking P2Y_13_-R (Figure 5A). Similarly, ex vivo assessment of lipolysis in WAT explants from mice fed HFSC revealed that the absence of P2Y_13_-R enhanced stimulated lipolytic activity in both iWAT and eWAT (Figure 5, B and C). Mechanistically, the absence of P2Y_13_-R in WAT resulted in heightened CL316,243-induced activation of hormone-sensitive lipase in WAT explants, evidenced by increased phosphorylation of hormone-sensitive lipase at Ser660 (Figure 5D). This correlated with a higher release of pro-inflammatory cytokines, including interleukin-6 (IL-6) and monocyte chemoattractant protein-1 (MCP-1), but not tumor necrosis factor-α (TNF-α), from WAT explants lacking P2RY_13_-R (Figure 5E).

### The absence of P2Y_13_-R alters lipoprotein metabolism and insulin sensitivity associated with chronic systemic inflammation.

Given that chronic elevation of FFA levels and production of adipocytokines promote metabolic disturbances such as dyslipidemia, insulin resistance, and low-grade inflammation, we next examined the effect of P2Y_13_-R deletion on lipid and glucose homeostasis, as well as systemic inflammation.

Compared with CD feeding, challenge with HFSC increased plasma levels of TGs and cholesterol, independent of P2Y_13_-R expression, except at 40 weeks of HFSC feeding, after which the lack of P2Y_13_-R worsened diet-induced hypercholesterolemia (Supplemental Figure 3, A and B). In terms of lipoprotein-related parameters, this higher level of total plasma cholesterol in the absence of P2Y_13_-R was associated with a higher cholesterol content in both LDL and HDL (Supplemental Figure 3C). With respect to lipoprotein particle subclasses (Supplemental Figure 3, D–F), the lack of P2Y_13_-R resulted in higher levels of all LDL particle subclasses (Supplemental Figure 3E) and large HDL particles (Supplemental Figure 3F).

**Figure 6:**
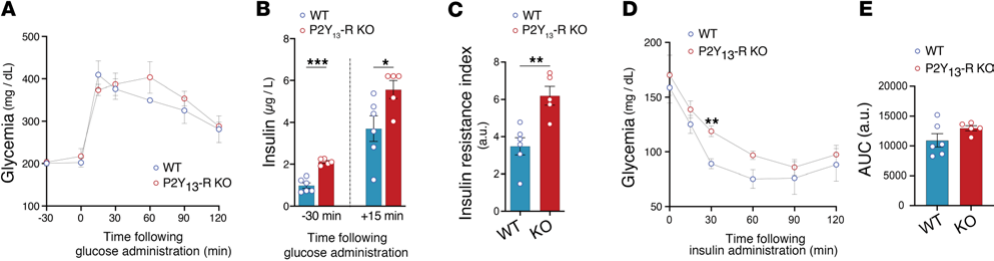
Glucose metabolism is altered by P2Y_13_-R deletion in mice fed an HFSC diet. (**A**) OGTT, 3 g/kg glucose (*n* = 5 or 6 mice per group). (**B**) OGTT-associated basal and glucose-stimulated insulinemia values in overnight-fasted mice (*n* = 5 or 6 mice per group). (**C**) Insulin resistance index based on OGTT-associated blood glucose and insulin values (*n* = 5 or 6 mice per group). (**D**) ITT, 1 U/kg insulin (*n* = 5 or 6 mice per group). (**E**) AUC for **D** (*n* = 5 or 6 mice per group). Open blue and red circles represent WT and P2Y_13_-R–KO mice, respectively. All data are expressed as mean ± SEM. *P < 0.05, **P < 0.01, ***P < 0.001 (**A** and **D**, 2-way ANOVA followed by Bonferroni’s post hoc test was used for genotype comparison; **B**, **C**, and **E**, 2-tailed unpaired Student’s *t* test was used for genotype comparison). Results were obtained from mice fed an HFSC diet for 8 weeks (**A**–**C**) and 10 weeks (**D** and **E**). OGTT, oral glucose tolerance test; HFSC, high-fat high-sucrose high-cholesterol; ITT, insulin tolerance test; KO, knockout; WT, wild-type.

Regarding glucose homeostasis, both WT and P2Y_13_-R–KO mice, fed on an HFSC for 8 weeks, displayed a significant increase in fasting plasma glucose and insulin when compared with mice fed a CD (Supplemental Figure 4). This indicates that the HFSC feeding efficiently impaired glucose metabolism. There was no difference in OGTT between WT and P2Y_13_-R–KO mice that were fed 8 weeks of HFSC diet (Figure 6A). However, the absence of P2Y_13_-R favored insulin resistance, which was evident from the increased basal and glucose-stimulated insulin levels (Figure 6B) and resulted in a higher insulin resistance index (Figure 6C). Accordingly, the ITT revealed that P2Y_13_-R–KO mice were less insulin tolerant, as evidenced by significantly reduced glucose clearance 30 minutes after insulin injection compared with the control group, though there was no significant difference in glucose AUC between the 2 groups (Figure 6, D and E). To further examine the impact of P2Y_13_-R deficiency on insulin sensitivity in WAT and liver, we injected HFSC-fed WT and P2Y_13_-R–KO mice with insulin (1 mU/g body weight, i.v.) and euthanized them 3 minutes later to rapidly collect tissue for insulin signaling analysis (28). The immunoblotting studies showed decreased insulin-mediated phosphorylation of Akt in the liver and eWAT in the absence of P2Y_13_-R (Supplemental Figure 5, A and C), indicating impaired insulin sensitivity in these tissues, with no such effect observed in iWAT (Supplemental Figure 5B).

**Figure 7:**
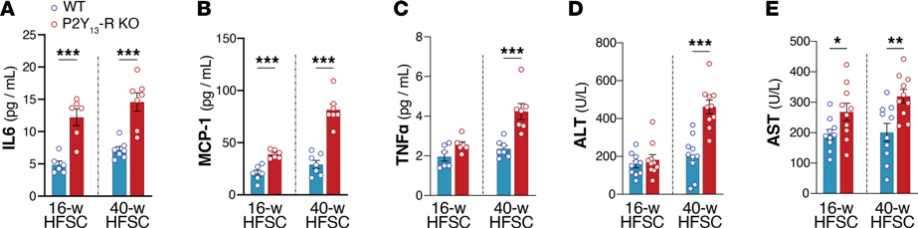
Lack of P2Y_13_-R increases systemic inflammation and plasma transaminases. (**A**) Plasma concentrations of IL-6 (*n* = 6 or 7 mice per group). (**B**) Plasma concentrations of MCP-1 (*n* = 6 or 7 mice per group). (**C**) Plasma concentrations of TNF-α (*n* = 6 or 7 mice per group). (**D**) Plasma concentrations of ALT (*n* = 10 mice per group). (**E**) Plasma concentrations of AST (*n* = 10 mice per group). Open blue and red circles represent WT and P2Y_13_-R–KO mice, respectively. All data are expressed as mean ± SEM. *P < 0.05, **P < 0.01, ***P < 0.001 (**A**–**E**, 2-tailed unpaired Student’s *t* test was used for genotype comparison). Results were obtained from mice fed an HFSC diet for 16 weeks (16-w) or 40 weeks (40-w). ALT, alanine aminotransferase; AST, aspartate aminotransferase; HFSC, high-fat high-sucrose high-cholesterol; IL-6, interleukin-6; KO, knockout; MCP-1, monocyte chemoattractant protein-1; TNF-α, tumor necrosis factor-α; WT, wild-type.

Dysregulation of lipolysis and insulin sensitivity in obesity often correlates with increased systemic inflammation. To examine P2Y_13_-R’s potential role in this process, we analyzed plasma levels of IL6, MCP-1, and TNF-α, as markers of systemic inflammation, along with plasma transaminases, as indicators of hepatic injury. We found that P2Y_13_-R deficiency in HFSC diet–fed mice increased systemic inflammation, as shown by increased plasma levels of IL-6, MCP-1, and TNF-α (Figure 7, A–C). Plasma levels of transaminases, alanine aminotransferase (ALT) and aspartate aminotransferase (AST), were also increased in P2Y_13_-R–KO mice fed HFSC diet, reflecting potential liver injury (Figure 7, D and E).

Together, these results indicate that HFSC diet challenge in mice lacking P2Y_13_-R alters lipoprotein metabolism, promotes insulin resistance, and increases systemic inflammation.

### The absence of P2Y_13_-R exacerbates diet-induced hepatic steatosis and fibrosis.

Increased plasma levels of FFAs and transaminases are associated with the development of MASLD, which ranges from hepatic steatosis to more advanced stages, including fibrosis and metabolic dysfunction-associated steatohepatitis (MASH, formally named NASH; ref. 4). Therefore, we analyzed whether hepatic steatosis and fibrosis, mediated by the HFSC diet, were worsened in P2Y_13_-R–KO mice.

**Figure 8:**
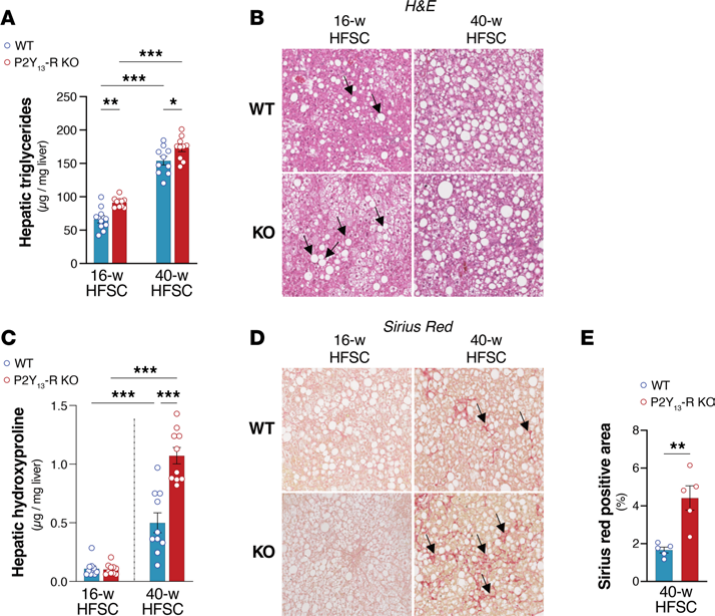
Lack of P2Y_13_-R promotes steatosis and aggravates liver fibrosis. (**A**) Hepatic concentrations of TG (*n* = 10 mice per group). (**B**) Representative H&E staining of liver sections (original magnification, 20×). Arrows indicate lipid droplets. (**C**) Hepatic concentrations of hydroxyproline (HYP) (*n* = 10 mice per group). (**D**) Representative Sirius red (SR) staining of liver sections (original magnification, 20×). Arrows indicate collagen fibers. (**E**) Quantification of SR-positive area in liver sections from **D** (*n* = 5 mice per group). Open blue and red circles represent WT and P2Y_13_-R–KO mice, respectively. All data are expressed as mean ± SEM. *P < 0.05, **P < 0.01, ***P < 0.001 (**A** and **C**, 1-way ANOVA followed by Bonferroni’s post hoc test was used for group comparison; **E**, 2-tailed unpaired Student’s *t* test was used for genotype comparison). Results were obtained from mice fed an HFSC diet for 16 weeks (16-w) or 40 weeks (40-w). HFSC, high-fat high-sucrose high-cholesterol; KO, knockout; TG, triglyceride; WT, wild-type.

Liver TG accumulation was measured as a hallmark of HFSC-mediated hepatic steatosis. Figure 8A shows that after 16 weeks on the HFSC diet, P2Y_13_-R–KO mice had significantly higher hepatic TG content than WT mice while the hepatic cholesterol content remained unchanged (Table 1). When the HFSC regimen extended to 40 weeks, hepatic TG content further increased but remained significantly higher in P2Y_13_-R–KO mice (Figure 8A). Analysis of lipid fluxes through the liver under HFSC diet showed that the absence of P2Y_13_-R did not affect the secretion rate of VLDL by the liver but reduced hepatic secretion of bile cholesterol, resulting in a significant decrease in fecal cholesterol content (Table 1). The exacerbation of diet-induced steatosis in P2Y_13_-R–KO mice compared with WT mice was also illustrated by the increased lipid droplets observed in H&E-stained liver sections (Figure 8B). Moreover, P2Y_13_-R–KO mice fed the HFSC diet for 16 weeks showed increased mRNA expression of genes involved in fatty acid handling and de novo lipogenesis, including fatty acid transporter (*Fat*/*Cd36*), fatty acid-binding protein 1, long-chain fatty acid transport protein 1, acetyl-CoA synthetase, elongation of long-chain fatty acids family member 6, and stearoyl-CoA desaturase-1 (Supplemental Figure 6A). Additionally, higher expression of inflammation-associated genes, such as *Tnfa*, *Mcp1*, and adhesion G protein–coupled receptor E1 (*Adgre1/F4/80*), was observed in these mice (Supplemental Figure 6B).

Hydroxyproline (HYP) content in the liver and Sirius red (SR) staining of liver sections were then used as indicators of the progression of hepatic fibrosis (29). After 40 weeks of HFSC feeding, mice developed liver fibrosis, with even more pronounced fibrosis observed in mice lacking P2Y_13_-R. This was evidenced by additional hepatic HYP content, SR staining in liver sections (Figure 8, C–E), and increased expression of genes related to fibrosis, including α–smooth muscle actin and *Tgfb1* (Supplemental Figure 7).

**Figure 9:**
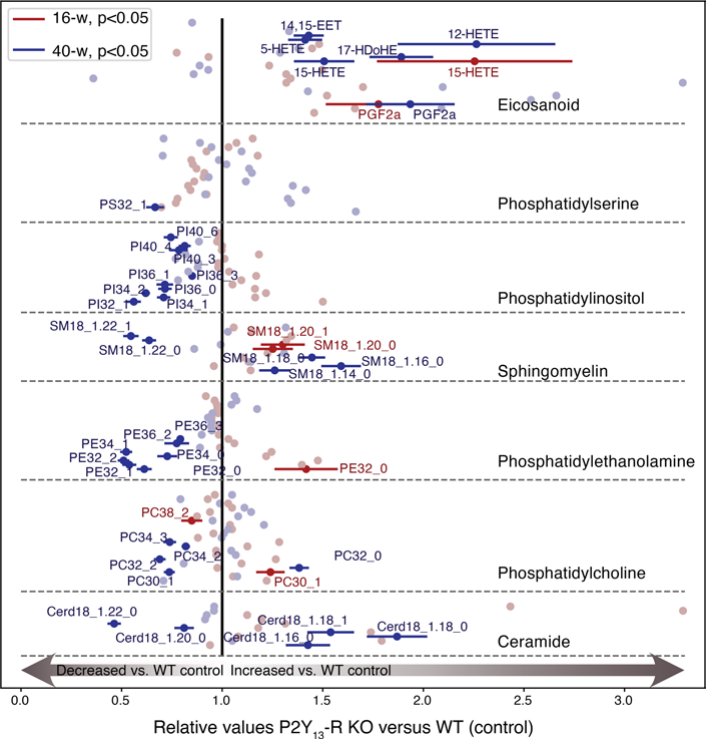
Lack of P2Y_13_-R impacts liver lipidome after 16 and 40 weeks of HFSC diet. A comparative analysis of the effects of 16 weeks (16-w) and 40 weeks (40-w) of HFSC diet in WT and P2Y_13_-R–KO mice was conducted regarding phospholipids and eicosanoids. Data are presented as the mean of the P2Y_13_-R–KO relative values to WT ± SEM. There were at least 5 mice per group at 16 weeks and 4 mice per group at 40 weeks. The significant results are labeled and color-coded depending on the duration of the diet. HETE, hydroxyeicosatetraenoic acid; HFSC, high-fat high-sucrose high-cholesterol; KO, knockout; PI, phosphatidylinositol; PE, phosphatidylethanolamine; PC, phosphatidylcholine; SM, sphingomyelin; WT, wild-type.

To better understand the impact of P2Y13-R deletion on the liver’s lipid profile in this scenario, we conducted a lipidomic analysis focusing on eicosanoids (EICs), phospholipids (PLs), and sphingolipids in liver samples obtained from P2Y13-R–KO and WT mice fed an HFSC diet for 16 and 40 weeks. These lipid classes have shown disturbed hepatic levels in various animal models of MASH, as well as in human cohorts of patients with MASH (30, 31). The results are presented in Figure 9. After 16 weeks of HFSC diet, only a few differences were observed, including an increase in 15-HETE) and prostaglandin F2 alpha (PGF2a) for EIC and SM species. However, after 40 weeks of HFSC diet, we observed substantial differences in several lipid species between P2Y13-R–KO and WT mice. Specifically, there was an increase in 5-, 12-, and 15-HETE; 14,15-epoxyeicosatrienoic acid; and PGF2a among EICs. Additionally, there was a decrease in PI, PE, and PC species in P2Y13-R–KO mice compared with WT mice. Ceramides showed changes in both directions, with species containing shorter fatty acid moiety (C16-18) increasing, while those with C20-22 fatty acid moiety decreased. Finally, a similar trend was observed for SM, with species containing C14-18 (phosphocholine group and fatty acid residue) increasing and those containing C22 decreasing. These changes demonstrate the substantial impact of P2Y13-R deficiency on liver lipidomics in the progression of MASLD.

### Contribution of P2Y_13_-R–mediated adipose tissue lipolysis to hepatic steatosis.

**Figure 10:**
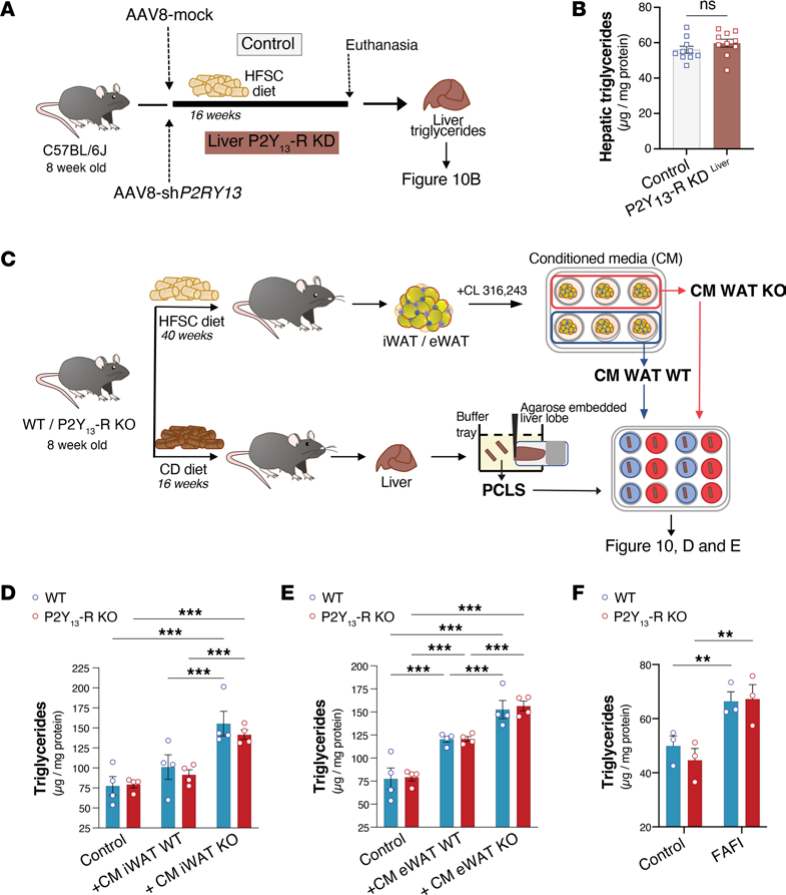
Increased liver steatosis associated with the lack of P2Y_13_-R depends on adipose tissue lipolysis. (**A**) Experimental design of liver-specific P2Y_13_-R–KD (P2Y_13_-R–KD^liver^) and control mice. (**B**) Hepatic TG concentration from P2Y_13_-R–KD^liver^ and control mice fed HFSC diet for 16 weeks (*n* = 10 mice per group). (**C**) Study design for precision-cut liver slice (PCLS) experiments. (**D** and **E**) TG concentrations in liver slices from chow diet–fed WT and P2Y_13_-R–KO mice in control condition or after 48 hours’ treatment with conditioned media originating from CL316,243-stimulated iWAT or eWAT explants originating from WT or P2Y_13_-R–KO mice that were HFSC fed for 40 weeks (*n* = 4 mice per group). (**F**) TG concentrations in liver slices from chow diet–fed WT and P2Y_13_-R–KO mice after 48 hours of treatment with FAFI media containing oleic acid, palmitic acid, insulin, and fructose. Open blue and red circles represent PCLS from WT and P2Y_13_-R–KO mice, respectively (*n* = 3 mice per group). All data are expressed as mean ± SEM. **P < 0.01, ***P < 0.001 (**B**, 2-tailed unpaired Student’s *t* test was used for genotype comparison; **D**–**F**, 2-way ANOVA followed by Bonferroni’s post hoc test was used for group comparison). CM, conditioned media; eWAT, epididymal white adipose tissue; FAFI, fatty acids, fructose and insulin; HFSC, high-fat high-sucrose high-cholesterol; iWAT, inguinal white adipose tissue; KD, knockdown; KO, knockout; ns, nonsignificant; TG, triglyceride; WT, wild-type.

To further examine the role of hepatic P2Y_13_-R in liver steatosis, we produced mice with liver-specific P2Y_13_-R knockdown (KD) using adeno-associated virus serotype 8 (AAV8), which is known to display a strong hepatic tropism in mice (32, 33). Mice were transduced with AAV8 carrying either mock (AAV8-mock) or shRNA targeted against P2Y_13_-R (AAV8-sh*P2RY13*) then fed with HFSC diet for 16 weeks to induce hepatic steatosis (Figure 10A). As shown in Supplemental Figure 8, AAV8-mediated KD of P2Y_13_-R successfully blocked *P2ry13* expression in liver while it remained unchanged in other tissues, indicating that liver-specific KD of P2Y_13_-R was maintained over the HFSC diet duration. Comparing AAV8-mock– and AAV8-sh*P2RY13*–transduced mice fed HFSC (control and P2Y_13_-R–KD^liver^, respectively), we observed no statistically significant difference in OGTT or in basal and glucose-stimulated insulin levels (Supplemental Figure 9, A and B) or in hepatic TG content (Figure 10B) or liver lipidome, with the exception of the level of EICs such as 5-, 12-, and 15-HETE and PGF2a that were increased in the liver of P2Y_13_-R–KD^liver^ mice (Supplemental Figure 10).

These observations suggest that the increase in HFSC-mediated insulin resistance and hepatic steatosis reported in P2Y_13_-R–KO mice (Figures 6 and 8) does not depend on P2Y_13_-R activity in the liver.

Further, to determine if the increased steatosis observed in HFSC-fed P2Y_13_-R–KO mice was originating from adipose tissue to liver crosstalk, we analyzed the effect of conditioned media from WAT on precision-cut liver slices (PCLS). This model preserves the original architecture and cellular composition of liver, allowing an organ-specific evaluation (34). We cultured PCLS from CD-fed WT or P2Y_13_-R–KO in conditioned media from CL316,243-stimulated eWAT or iWAT explants originating from WT or P2Y_13_-R–KO mice fed HFSC diet for 40 weeks (Figure 10C). There was a significant increase in FFA content in media from P2Y_13_-R–KO mice compared with that from WT mice (Figure 5, B and C). Regardless of P2Y_13_-R expression in PCLS, their TG content increased when incubated with conditioned media derived from P2Y_13_-R–KO mice compared with conditioned media from WT mice (Figure 10, D and E). Additionally, when PCLS were exposed to media containing a defined concentration of fatty acids, fructose, and insulin (FAFI), a similar increase in TG content was observed for PCLS derived from P2Y_13_-R–KO or WT mice (Figure 10F).

Overall, these data suggest that the increased liver steatosis induced by HFSC feeding in P2Y_13_-R–KO mice could be driven by heightened adipose tissue lipolytic activity rather than by the absence of P2Y_13_-R in the liver.

## Discussion

Chronic unrestrained adipose tissue lipolysis, as observed in obesity, leads to peripheral insulin resistance and the accumulation of fat in nonadipose tissues, predominantly in the liver (3). In adipocytes, lipolysis is primarily regulated by GPCRs, which elicit either lipolytic or antilipolytic effects via G_s_ or G_i_ coupling, respectively. G_s_-mediated activation of AC increases intracellular cAMP levels, activating PKA, which phosphorylates and activates hormone-sensitive lipase. Conversely, G_i_ coupling inhibits AC, reducing cAMP level and suppressing lipolysis (1).

Although the anti- or lipolytic action of certain adipocyte GPCRs, such as α_2_- and β-ARs responding to catecholamines, are well defined, as is the antilipolytic effect of metabolites like nicotinic acid on the HCA2 receptor (also known as GPR109a), the role of most adipose tissue GPCRs in regulating lipolysis remains underexplored. The significance of the G_i_ signaling pathway in restraining adipose tissue lipolysis has been recently highlighted. In particular, a study reported that mice lacking functional G_i_ proteins in adipocytes displayed increased lipolysis, resulting in reduced insulin sensitivity and higher hepatic steatosis when maintained on an obesogenic diet (35).

In this study, we found that G_i_-coupled P2Y_13_-R activity regulates adipocyte lipolysis. Primary adipocytes from P2Y_13_-R–KO mice, in both inguinal and epididymal depots, displayed increased intracellular cAMP levels and lipolytic activity compared with control mice, indicating a negative effect of P2Y_13_-R signaling on lipolysis.

Consistent with this finding, when P2Y_13_-R–KO mice were fed a Western-style diet (HFSC) to induce metabolic disturbances associated with obesity (27), they displayed higher levels of β-AR–stimulated lipolysis in WAT explants and in vivo. This increase was associated with higher levels of FFA secretion and circulating levels, respectively, and more severe insulin resistance compared with control littermates. Moreover, under these conditions, P2Y_13_-R–KO mice exhibited diminished hepatic insulin sensitivity and more severe liver steatosis than their control counterparts. Additionally, with prolonged HFSC feeding to induce hepatic fibrosis, fibrosis was significantly more pronounced in P2Y_13_-R–KO mice compared with controls. This phenotype was linked to elevated levels of hepatic HETE, a class of EICs generated by arachidonic acid oxidation. Some HETEs whose levels were increased in P2Y_13_-R–KO mice, including 5-, 12-, and 15-HETE, have been reported as lipid signatures of MASLD progression (36, 37). Additionally, the increase in hepatic saturated C16 ceramide is of particular interest, as prior studies have revealed its involvement in metabolic syndrome and MASLD development through its production by ceramide synthase 6 (38, 39). Finally, P2Y_13_-R deficiency was associated with reduced hepatic levels of several long-chain PL species (PI, PE, PC), which have been shown to decline with MASLD progression in patients, compared with healthy individuals (30).

Using P2Y_13_-R–KD^liver^ mice, we have shown that the increased hepatic steatosis and insulin resistance observed in P2Y_13_-R–KO mice could not be attributed to the lack of P2Y_13_-R in the liver. Instead, when liver slices were treated with adipose tissue media from P2Y_13_-R–KO mice, there was a marked increase in steatosis compared with treatment with adipose tissue media from WT mice, regardless of P2Y_13_-R expression in the liver. These findings suggest that heightened adipose tissue lipolytic activity in P2Y_13_-R–KO mice may be a key factor contributing to the increased liver steatosis (i.e., TG accumulation). However, it is noteworthy that certain EICs were more elevated in the livers of both P2Y_13_-R–KO and P2Y_13_-R–KD^liver^ mice compared with their respective control groups. Considering that EIC species primarily discriminate between cirrhosis and other MASLD stages (36), this observation suggests a potential involvement of hepatic P2Y_13_-R in mitigating the progression of MASLD, independent of the role played by adipocyte P2Y_13_-R in protecting against hepatic steatosis.

In hepatocytes, P2Y_13_-R signaling promotes HDL endocytosis (25), with the cholesterol within HDL eventually being secreted into the bile (40, 41). This process substantially contributes to the overall removal of excess cholesterol from the body, offering protection against atherogenesis in atherogenic ApoE-KO mice (42). While we have excluded P2Y_13_-R’s contribution to protecting against liver steatosis against liver steatosis, its role in regulating liver cholesterol catabolism could confer protection against the subsequent development of hepatic fibrosis. This hypothesis is supported by studies emphasizing hepatic cholesterol’s pivotal role as a lipotoxic molecule in MASLD development (43, 44). Nonetheless, in our model of diet-induced metabolic disorders, the absence of P2Y_13_-R does not affect hepatic cholesterol content. Instead, it results in increased plasma cholesterol levels, mainly HDL-cholesterol, correlating with decreased hepatobiliary cholesterol excretion. These observations strongly suggest that P2Y_13_-R is more likely involved in regulating the flux of cholesterol through the liver, rather than maintaining steady-state hepatic cholesterol concentrations.

In addition to the P2Y_13_-R, 2 other P2Y receptors have been identified to play roles in regulating lipolysis in adipocytes. The first is P2Y_2_-R, primarily activated by ATP or UTP, and the second is P2Y_14_-R, activated by UDP or UDP-glucose (17, 45). P2Y_2_-R activity in adipocytes suppresses lipolysis through G_q_ signaling, inhibiting Ca^2+^-sensitive AC isoforms (17). However, the in vivo consequences of its adipocyte function have not been assessed yet. Similar to P2Y_13_-R, P2Y_14_-R exerts antilipolytic activity in adipocytes through G_i_ signaling (45). P2Y_14_-R shares 40%–45% amino acid sequence identity with P2Y_13_-R, and its gene is located at the same chromosomal location as *P2RY13*, specifically at 3q25.1 of human chromosome 3 (46). Mice lacking P2Y_14_-R in adipocytes exhibit enhanced lipolysis in the fasted state and are protected from high-fat diet–induced (HFD-induced) obesity because of decreased fat mass (45). Surprisingly, these mice are also protected from liver steatosis and present improved glucose tolerance and insulin sensitivity (45). While this unexpected phenotype contrasts with the conventional notion that increased lipolysis could lead to ectopic fat accumulation and development of T2D (2, 3), the authors hypothesized that the reduced obesity, coupled with a slight enhancement of energy expenditure, observed in HFD-fed mice lacking adipocyte P2Y_14_-R, may contribute to an overall improvement in whole-body metabolism. Collectively, these studies, including the one presented in this work, highlight the involvement of various purinergic signaling pathways in the lipolytic activity of adipose tissue and underscore the complex interplay between purinergic signaling pathways and the communication between adipose tissue and the liver in the regulation of lipolysis and hepatic steatosis.

The current study has strengths and limitations. First, further research is needed to precisely ascertain the contribution of the adipocyte P2Y_13_-R’s lipolytic activity to MASLD regulation through the intricate crosstalk between adipose tissue and the liver. However, we have documented an increase in diet-induced metabolic dysfunctions in whole-body P2Y_13_-R–deficient mice, adding to the atheroprone phenotype previously reported in these mice (42, 47). These results suggest that pharmacological activation of P2Y_13_-R could provide protection against cardiovascular and metabolic diseases.

Second, further validation of the clinical relevance of our findings is needed. Nonetheless, support for translatability to humans has been gained from the observed negative correlation between the adipose expression of *P2RY13* and adipocyte lipolytic activity in humans. Additionally, we have previously identified a genetic variant of *P2RY13* (rs3732757) associated with cardioprotection (48). Together, these clinical data suggest P2Y_13_-R’s involvement in cardiovascular and metabolic traits in humans, though further confirmation is required to conclusively affirm the translatability of our findings to humans.

In conclusion, P2Y_13_-R signaling appears to protect against metabolic dysfunctions associated with obesity. Its potential to mitigate MASLD development might be secondary to its role in inhibiting adipose tissue lipolysis. These findings prompt further investigations into the intricate molecular mechanisms governing the adipose/liver axis and how purinergic signaling networks modulate this crosstalk. Our findings provide a rational basis for the development of P2Y_13_-R agonists for treating metabolic diseases linked to obesity, including MASLD and T2D.

## Methods

### Sex as a biological variable.

Our study exclusively examined male mice. It is unknown whether the findings are relevant for female mice.

### P2ry13 gene expression and lipolysis measures in human samples.

The expression of the *P2ry13* gene was determined using gene microarray data from subcutaneous WAT in a cohort of women, including 30 participants with obesity (BMI > 30 kg/m^2^) and 26 without (BMI < 30 kg/m^2^) (49). Adipocytes were isolated, then treated with increasing concentrations of isoprenaline for 2 hours, and lipolysis was measured by glycerol release into the medium (50).

### Animals and study design.

P2Y_13_-R–deficient (P2Y_13_-R^–/–^) mice on a C57BL/6J background were generated as previously described (40). P2Y_13_-R–KO and P2Y_13_-R^+/+^ littermate (WT) mice were housed at 18°C–24°C, with a 12-hour light/12-hour dark schedule. They were provided with ad libitum access to food and water. Only male mice were used in this study. The purpose of the animal study was to investigate the effects of P2Y_13_-R on adipose tissue lipolysis, diet-induced insulin resistance, and fatty liver disease. Following a 3-week weaning period, mice were fed a regular diet (chow, V1534, ssniff) until they reached 8 weeks of age. Subsequently, groups were randomly assigned using the online random number generator from GraphPad (https://www.graphpad.com/quickcalcs/randomize1/) to either continue on the regular diet for 16 weeks or, in other group conditions, switch to an HFSC diet (TD.88137, Envigo, containing 42% kcal from fat, 0.2% cholesterol, and 34% sucrose by weight) for either 16 or 40 weeks (Figure 4, study design).

### Primary adipocyte and explant isolation from WAT.

For isolated adipocytes, adipocytes were isolated from iWAT and eWAT after collagenase digestion. The digestion was performed for 30 minutes at 37°C in a Krebs-Ringer bicarbonate buffer (pH 7.4) containing 0.5 mM CaCl_2_, 1 M HEPES, 10 mM glucose, 3.5% BSA, and 1 mg/mL collagenase (C-6885, MilliporeSigma). At the end of digestion, the fat cell suspension was filtered and rinsed 3 times. For explants, iWAT and eWAT were carefully dissected and minced. A total of 50 mg of tissue was incubated 90 minutes in a 6-well plate containing Krebs-Ringer bicarbonate buffer (pH 7.4) with 0.5 mM CaCl_2_, 1 M HEPES, and 3.5% BSA.

### Intracellular cAMP assay.

Primary adipocytes isolated from eWAT or iWAT were seeded in a 384-well plate, and intracellular cAMP content was measured using an HTRF-based cAMP-Gi kit (62AM9PEB, Cisbio) according to manufacturer’s instructions.

### In vitro lipolysis on isolated adipocytes or explants.

Primary adipocytes were isolated as previously described, and 100 μL were incubated for 90 minutes in glass tube containing 1 mL of Krebs-Ringer bicarbonate buffer (pH 7.4) containing 0.5 mM CaCl_2_, 1 M HEPES, 10 mM glucose, and 3.5% BSA alone or with 10^–10^ to 10^–6^ M CL316,243 (C5976, MilliporeSigma), 10^–6^ M isoprenaline (I5627, MilliporeSigma), 10^–6^ M FSK (MilliporeSigma F6886), or 10^–6^ M hormone-sensitive lipase inhibitor BAY (NoValix), depending on the experiment. Similar conditions were used for adipose tissue explants. Lipolysis was evaluated by measuring glycerol and FFA concentrations in cell or explant supernatants with commercial kits according to manufacturer’s instructions (Free Glycerol Reagent, F6428, MilliporeSigma, and NEFA FS, DiaSys Diagnostic System, respectively). Results were normalized by the number of adipocytes or the weight of explants, respectively.

### In vivo lipolysis.

The mice were subjected to a 6-hour fasting period, after which they received an intraperitoneal injection of the β_3_-AR agonist CL316,243 (1 mg/kg body weight, C5976, MilliporeSigma). Plasma samples were collected 15 minutes after the administration of CL316,243 in order to measure glycerol and FFA levels.

###  Quantification of inflammatory cytokines in plasma and explant culture medium.

Plasma and culture medium levels of TNF-α and MCP-1 were determined using ELISAs (BioLegend, 430901 and 431301, respectively) in accordance with the manufacturer’s instructions. For the measurement of IL-6 levels, a Luminex magnetic bead–based immunoassay (MilliporeSigma) was conducted following the manufacturer’s instructions.

### OGTT and insulin resistance index determination.

After 8 weeks of HFSC diet, mice were fasted overnight and received an oral glucose load (3 g/kg body weight). Glycemia was measured by tail vein sampling with portable glucometer (Accu-check, Roche) 30 minutes before oral glucose load and at 0, 15, 30, 45, 60, 90, and 120 minutes after glucose load. Plasma insulin concentration was determined 30 minutes before and 15 minutes after glucose loading using an ELISA kit (Mercodia) according to the manufacturer’s instructions. The insulin resistance index was calculated by multiplying the AUC of both glycemia (–30 to 120 minutes) and plasma insulin (–30 to 15 minutes) and was expressed in arbitrary units.

### ITT.

After the mice underwent 10 weeks of HFSC diet, overnight-fasted mice were intraperitoneally injected with insulin (1 mU/g, Actrapid; Novo Nordisk A/S). Blood was collected from the tail vein at time 0 and after 15, 30, 45, 60 minutes for glycemia determination.

### Tissue protein extraction and Western blot analysis.

Tissue samples were homogenized using the Precellys lysing kit in RIPA buffer (50 mM Tris-HCl, pH 7.4, 150 mM NaCl, 1% NP-40, 0.5% sodium deoxycholate, 0.1% sodium dodecyl sulfate) or lysis buffer (50 mM Tris, pH 7.4, 500 mM NaCl, 1% NP-40, 20% glycerol, 5 mM EDTA, and 1 mM phenylmethylsulfonyl fluoride), respectively, supplemented with complete protease inhibitor cocktail and phosphatase inhibitor cocktail (Roche). The tissue lysates were then centrifuged at 16,000*g* for 10 minutes at 4°C, and the protein concentration in supernatants was measured using the Bradford method (Bio-Rad). The protein lysates were denatured at 90°C for 10 minutes and then separated by 4%–12% SDS-PAGE (Invitrogen, Thermo Fisher Scientific) and transferred onto nitrocellulose membranes (Bio-Rad). The membranes were incubated overnight at 4°C with primary antibody prepared in 1× TBS with 5% w/v BSA and 0.1% Tween 20 (dilution 1:1,000). On the next day, the membranes were washed and incubated with HRP-conjugated anti-rabbit or anti-mouse secondary antibodies (dilution 1:5,000). Specific immunoreactive bands were visualized and quantified using SuperSignal West Pico Chemiluminescent Substrate (Pierce, Thermo Fisher Scientific) and ChemiDoc Touch Imaging System (Bio-Rad). A list of the antibodies used is provided in Supplemental Table 1.

### Hormone-sensitive lipase phosphorylation.

Explants from eWAT and iWAT were placed in a 6-well plate containing Krebs-Ringer bicarbonate buffer (pH 7.4) with 0.5 mM CaCl_2_, 1 M HEPES, and 3.5% BSA and were stimulated for 90 minutes with 10^–6^ M CL316,243. Adipose tissue explants were then snap-frozen in liquid nitrogen for Western blot studies.

### In vivo insulin sensitivity.

P2Y_13_-R–KO and WT mice fed an HFSC diet for 10 weeks were fasted for 4 hours. Mice were then anesthetized (ketamine, 100 mg/kg, Rompun, Bayer; and xylazine, 15 mg/kg, Imalgene 1000, Merial) and received insulin at 1 mU/g of body weight into the portal vein (28). At 3 minutes postinjection, mice were euthanized, and liver, iWAT, and eWAT were rapidly harvested and snap-frozen in liquid nitrogen for Western blot studies.

### Liver total cholesterol and TG content.

A part of left liver tissue lobe (100 mg) was homogenized in 900 μL of phosphate buffer pH 7.4 with a tissue lyser. Lipids were extracted by mixing 125 μL of liver homogenate with 1 mL of CHCl_3_:MeOH (2:1). After centrifugation at 12,000*g* for 10 minutes, the chloroform phase was separated and evaporated under nitrogen flux; the dried residue was solubilized in 200 μL of isopropanol. TGs and cholesterol were measured using commercial kits based on CHOD-PAP and GPO-PAP detection method (Biolabo SA). Results were expressed as μg lipids/mg liver.

### Liver HYP content.

Hepatic HYP was determined from 250 to 350 mg of liver tissue, which was hydrolyzed in 5 mL of HCl 6N overnight at 110°C. The samples were diluted in citric-acetate buffer and treated with Chloramine T (857319, MilliporeSigma) and 4-(dimethyl)aminobenzaldehyde (156477, MilliporeSigma). The absorbance of the samples was measured at 550 nm. Results are presented as μg HYP/mg tissue.

### Liver histological analysis.

Parts of the left lobe were paraformaldehyde-fixed, paraffin-embedded, and sliced into 5 μm sections, which were stained with H&E or SR for histopathological analysis. For H&E staining, sections were incubated for 30 minutes in Mayer hematoxylin solution, rinsed 5 minutes in distilled water, and incubated in saturated lithium carbonate solution for 15 seconds, rinsed 3 minutes again in distilled water, and finally placed in 0.5% alcoholic eosin solution for 30 seconds. For SR staining, sections were incubated for 10 minutes in 1% SR (MilliporeSigma, France) dissolved in saturated picric acid and rinsed in distilled water. For all staining, sections were dehydrated for 15 minutes with absolute ethanol and cleared with Histoclear clearing agent (Euromedex) before mounting with DPX and coverslipping. The image acquisition was performed using the Pathology Slide Scanners NanoZoomer from Hamamatsu. The quantification of the liver fibrosis was measured from SR-stained sections using the Positive Pixel Count Algorithm of the software Aperio ImageScope from Leica Biosystems. The calculation was based on the percentage of positive pixels from collagen fibers related to the total pixels corresponding from all liver tissue surface.

### RNA extraction and real-time quantitative PCR analysis.

Total RNAs were prepared from tissues using the QIAzol lysis reagent (QIAGEN). Extracted RNAs were suspended in DNase/RNase-free water, and the concentration of each sample was measured at a wavelength of 260 nm on NanoDrop (Thermo Fisher Scientific). Reverse transcription of mRNA was performed using M-MLV Reverse Transcriptase (Thermo Fisher Scientific) in the presence of primer p(dT) (Promega) and RNase OUT (Thermo Fisher Scientific). Briefly, 1 μg of total RNA from each sample was used with 2 μL of random primers (500 μg/mL, Promega) and 0.5 μL RNAse OUT (Thermo Fisher Scientific). After 8 minutes of incubation at 75°C to inactivate DNase, 200 units of M-MLV Reverse Transcriptase was added, and the mixture was incubated at 25°C for 10 minutes and then at 37°C for 1 hour. The reverse transcription reaction was ended by heating the mixture at 95°C for 5 minutes. It was then chilled and stored at −80°C until use. Real-time quantitative PCR analysis was performed using SsoFast EvaGreen Supermix (Bio-Rad). PCR reactions were run in a 96-well format with 20 μL reaction mixture containing SsoFast EvaGreen (10 μL), cDNA from the reverse transcription reaction (2 μL), gene-specific primers (0.5 μL), and DNase/RNase-free water (7 μL). Reactions were run with a standard 2-step cycling program, 95°C for 3 seconds and 60°C for 30 seconds, for 40 cycles. mRNA expression values were obtained by the 2-^ΔΔCt^ method, where geometric mean of *Rps29* is used as the reference gene expression values. Primer sequences are listed in Supplemental Table 2.

### Analysis of the liver lipidome.

Liver tissue (50 mg) was snap-frozen in liquid nitrogen and stored at –80°C until lipid extraction. Samples were homogenized in 1 mL of methanol:EGTA 5 mM (2:1 v/v). From each homogenate, 3 separate aliquots were taken to perform PL and EIC extraction and protein content determination (Bradford method). Internal standards were added to each samples: for PL we used ceramide 18:1/12:0, PE 12:0/12:0, PC 13:0/13:0, SM 18:1/12:0, PI 14:1/17:0, and PS 12:0/12:0, whereas for EIC we used a mix of deuterium-labeled compounds (Lipoxin A4-d5, Leukotriene D4, 5S- hydroxy- 6E,8Z,11Z,14Z- eicosatetraenoic- 5,6,8,9,11,12,14,15- d8 acid).

For PL analysis, the equivalent of 2 mg of tissue was collected and extracted in CH_2_Cl_2_:MeOH:H_2_O (2.5: 2.5: 2.1, v/v/v) with 2% acetic acid. After centrifugation, the organic phase was collected, dried under nitrogen, and then dissolved in 50 μL of MeOH.

For EIC analysis, the equivalent of 10 mg of tissue was collected and mixed with 500 μL of HBSS and 260 μL of cold MeOH. After centrifugation, supernatants were transferred in a 96-well deep plate, and volume was adjusted to 2 mL with distilled water. Samples were then submitted to solid-phase extraction using Oasis HLB 96-well plate (30 mg/well, Waters) according to the manufacturer’s instructions. After extraction, the samples were dried under nitrogen and reconstituted in 10 μL of MeOH. All samples were analyzed by liquid chromatography-tandem mass spectrometry as previously described (52), and results were expressed as relative quantity for PL and as pg/mg proteins for EIC. Results are presented in a scatterplot generated in Python with the matplotlib library.

### Liver slice experiments.

All mice were sacrificed by cervical dislocation, and livers were collected in ice-cold HBSS (H6648, Merck). The lobes were separated, and the left and middle lobes were individually embedded in 3% agarose just before slicing. PCLS were prepared with a Compresstome VF-310-0Z (Precisionary). The embedded lobes were placed in a tray filled with ice-cold Krebs-Henseleit buffer supplemented with 25 mM d-glucose (Merck), 25 mM NaHCO_3_ (Merck), and 10 mM HEPES (Merck), saturated with carbogen (95% O_2_/5% CO_2_) and pH 7.42. The thickness of the slices was set at 170 μm. PCLS were placed in 12-well plates filled with Williams Medium E supplemented with l-glutamine (Merck) and gentamicin (Merck) or conditioned media from adipocytes. The plates were placed on an orbital shaker (set at 90 rpm) inside a cell culture incubator (37°C, 20% O_2_ and 5% CO_2_). The culture medium was refreshed after 24 hours, and the samples were collected after 48 hours of culture. Depending on the experiments, PCLS were treated with conditioned media from WAT explants from P2RY_13_-R–KO or WT mice or FAFI (250 μM oleic acid, 125 μM palmitic acid, 1 mM fructose, and 1 nM insulin).

### Adenovirus production and injection.

The shRNA sequence targeting the mouse P2Y_13_-R, AGCTTTTCCAAAAAATCCTTTCCGACTCACACCTCTCTTGAAGGTGTGAGTCGGAAAGGATGGT, was subcloned into pAAV8 plasmid (Addgene plasmid 112864) with H1 promoter, as previously described (47). The adenovirus expressing the P2RY13 shRNA (AAV8-sh*P2RY13*) and the mock adenovirus consisting of the vacant pAAV8-H1 vector (AAV8-mock) were produced by the Vectorology platform of Toulouse (Vectoul, CRCT-UMR 1037 INSERM/Université de Toulouse III/CNRS, Toulouse, France), with 2 × 10^13^ and 4 × 10^13^ vector genomes (vg)/mL, respectively. Male 8-week-old C57BL/6J mice (Envigo) were injected in the tail vein with 4 × 10^9^ vg. These mice were fed with HFSC diet for 16 weeks.

### Statistics.

Statistical analyses were performed using GraphPad Prism V.9.1.0 (GraphPad Software). The data were presented as the mean ± SEM for the indicated number of observations. Prior to conducing specific statistical tests, we assessed normality of variance. When appropriate, the data were log-transformed to achieved normal distribution. The analyses involved simple linear regression, 1- or 2-way ANOVA followed by the indicated post hoc test, or 2-tailed unpaired Student’s *t* test. The specific statistical tests that were used are indicated in the figure legends. The level of significance was defined as *P* < 0.05.

### Study approval.

The human study was approved by the ethical committee at the Karolinska University Hospital. All participants were informed in detail about the studies, and written informed consent was obtained. All procedures involving animals were performed in accordance with the principles and guidelines established by the National Institute of Health and Medical Research (INSERM) and were approved by the local Animal Care and Use Committee (CEEA122, Toulouse, France) and the French Ministry of Higher Education, Research and Innovation (APAFIS12359-2017110910398931). The investigation conforms to the directive 2010/63/EU of the European parliament.

### Data availability.

Values for all data points found in graphs are in the Supporting Data Values file.

## Author contributions

NV, PA, MR, DL, NS, CC, SN, and LOM conceived and designed this study. TD, EG, GC, DB, JPDS, VB, AV, and MN conducted experiments and acquired and analyzed data. MAM and GT assisted with experiments. TD, EG, and LOM drafted the manuscript and assembled figures. All authors discussed the results and commented on the manuscript.

## Supplementary Material

Supplemental data

Unedited blot and gel images

Supporting data values

## Figures and Tables

**Table 1:**
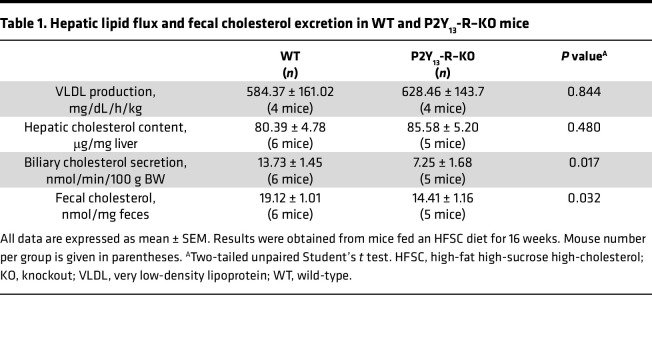
Hepatic lipid flux and fecal cholesterol excretion in WT and P2Y_13_-R–KO mice

## Version 1 03/12/2024

In-Press Preview

## Version 2 04/22/2024

Electronic publication
