# Supplementary material for: P2Y13 receptor deficiency favors adipose tissue lipolysis and worsens insulin resistance and fatty liver disease
Source: JCI Insight. 2024 Mar 12;9(8):e175623. doi: 10.1172/jci.insight.175623 (PMC11141875; doi:10.1172/jci.insight.175623)
Supplement: Supplemental data [file jciinsight-9-175623-s162.pdf]

Supplemental Data

Supplemental Table 1: List of antibodies used for Western blot studies.

Antibodies	Sources[a]	Catalog #
T-HSL	Cell Signaling	4107
p-HSL (Ser660)	Cell Signaling	4126
Akt	Cell Signaling	2920
p-Akt (Ser473)	Cell Signaling	9271

[a]Cell Signaling Technology, Leiden, The Netherlands, https://www.cellsignal.com/.

Supplemental Table 2: List of qPCR primer pair sequences and GeneBank accession numbers specific to each gene.

Gene symbol	Forward primer (5'-3')	Reverse primer (5'-3')	GeneBank
Acaca (*Acc1*)	GTTGAGACGCTGGTTTGTA	GGTCCTTATTATTGTCCCAGACGTA	NM_133360
Acsl1 (*Acs1*)	TCTGCCACAGTGCTGACGTTTC	TGTCCGTAGCCTTCATAGAACTGG	NM_007981
Adgre1 (*F4/80*)	TGACAACCAGACGGCTTGTG	GCAGGCGAGGAAAAGATAGTGT	NM_010130
Col1a1	TGCTTTCTGCCCGGAAG	GGGATGCCATCTCGTCCA	NM_007742
Elovl6	TCTGATGAACAAGCGAGCCA	TGGTCATCAGAATGTACAGCATGT	NM_130450
Fat (*Cd36*)	GCGACATGATTAATGGCACAGACG	TCCGAACACAGCGTAGATAGACC	NM_007643
Fabp1	ACCCAAAGTGGTCCGCAATGAG	TTCCAGCTTGACGACTGCCTTGAC	NM_017399
Fasn	CGGCTGCGTGGCTATGATTATG	GCAGCTTGCCTTGTTCACCTTC	NM_007988
Fatp1 / Slc27a1	ATTGCCAACATGGACGGCAAGGTC	ACATGCGTGAGGATACGGCTGTTG	NM_011977
Mcp1 / Ccl2	GCAGTTAACGCCCCACTCA	CCCAGCCTACTCATTGGGATCA	NM_011333
P2ry1	CGTGCTGGTGTGGCTCATT	CGAGTCCCAGTGCCAGAGTAG	NM_008772
P2ry12	TGTGGGCGTACCCTACAGAAAC	AGTGGAACTTGCAGACTGGCATC	NM_027571
P2ry13	TCAAAATCCTTTCCGACTCACA	GAGGAGAGCGTGCACACAAA	NM_028808
Rps29	GTCTGATCCGCAAATACGGG	AGCCTATGTCCATCGCGTACT	NM_009093
Scd1	CGTGGGCGCGGTGAT	CAACACCATGGCGTTCCA	NM_009127
aSMA	GTCCCAGACATCAGGGAGTAA	TCGGATACTTCAGCGTCAGGA	NM_007392
Tnfa	TGGGACAGTGACCTGGACTGT	TTCGGAAAGCCCATTTGAGT	NM_013693
Tgfb1	GAGCCCGAAGCGGACTACTA	CACTGCTTCCCGAATGTCTGA	NM_011577

Acc1, Acetyl-CoA carboxylase 1; *Acsl1*, Acyl-CoA synthetase long chain family member 1; *Adgre1* (*F4/80*), adhesion G protein-coupled receptor E1; *Col1a1*, collagen type I alpha 1; *Elovl6*, Elongation of long-chain fatty acids family member 6; *Fabp*1, Fatty acid-binding protein 1; *Fasn*, Fatty acid synthase; *Fat*, Fatty acid transporter; *Fatp1*, Long-chain fatty acid transport protein 1; *Mcp1*, monocyte chemoattractant protein-1; *Rps29*, Ribosomal protein S29; *Scd1*, Stearoyl-CoA desaturase-1; *aSMA*, alpha-smooth muscle actin; *Tnfa*, Tumor necrosis factor alpha; *Tgfb1*, transforming growth factor beta 1.

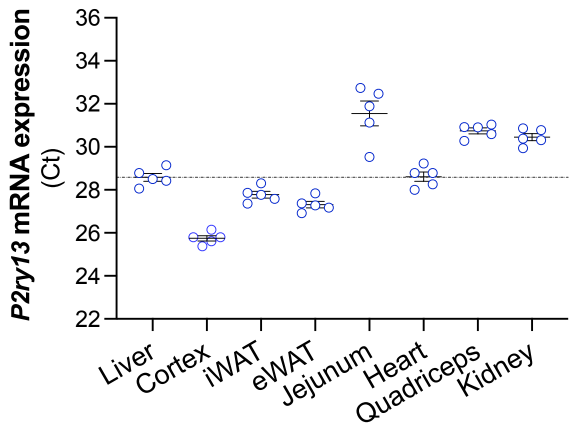

Supplemental Figure 1. Ct values for *P2ry13* gene expression in mouse tissue

All results were obtained from 24-month-old mice fed a chow diet (n = 5 mice).

Ct, cycle threshold, eWAT, epididymal white adipose tissue; iWAT, inguinal white adipose tissue

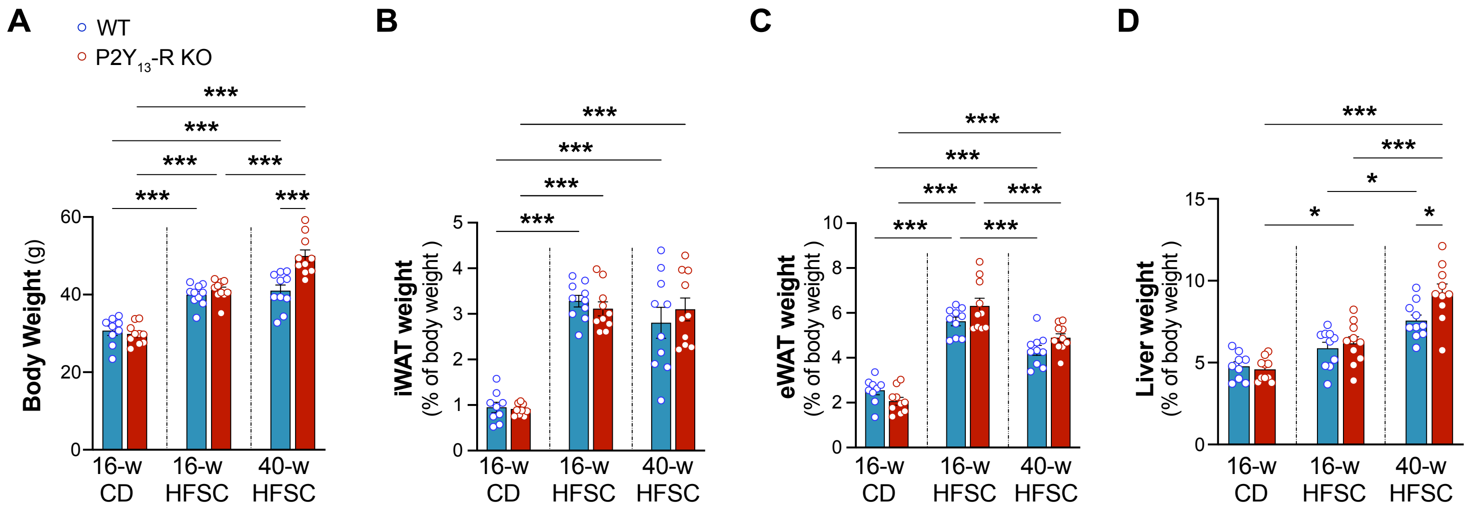

Supplemental Figure 2. Body weight and tissue mass changes throughout experiments.

(A) Body weight (n = 9 or 10 mice per group).

(B) iWAT weight (n = 9 or 10 mice per group)

(C) eWAT weight (n = 9 or 10 mice per group)

(D) liver weight (n = 9 or 10 mice per group).

Open blue and red circles represent WT and $P2Y_{13}$-R KO mice, respectively.

All data are expressed as mean ± SEM.

*P<0.05, ***P<0.001 (**A-D**, 1-way ANOVA followed by Holm-Sidak post hoc test was used for group comparison).

Results were obtained from mice fed a chow diet for 16-weeks (16-w), or HFSC diet for 16- or 40-weeks (40-w).

CD, chow-diet; eWAT, epididymal white adipose tissue; HFSC, high-fat high-sucrose high-cholesterol;

iWAT, inguinal white adipose tissue.

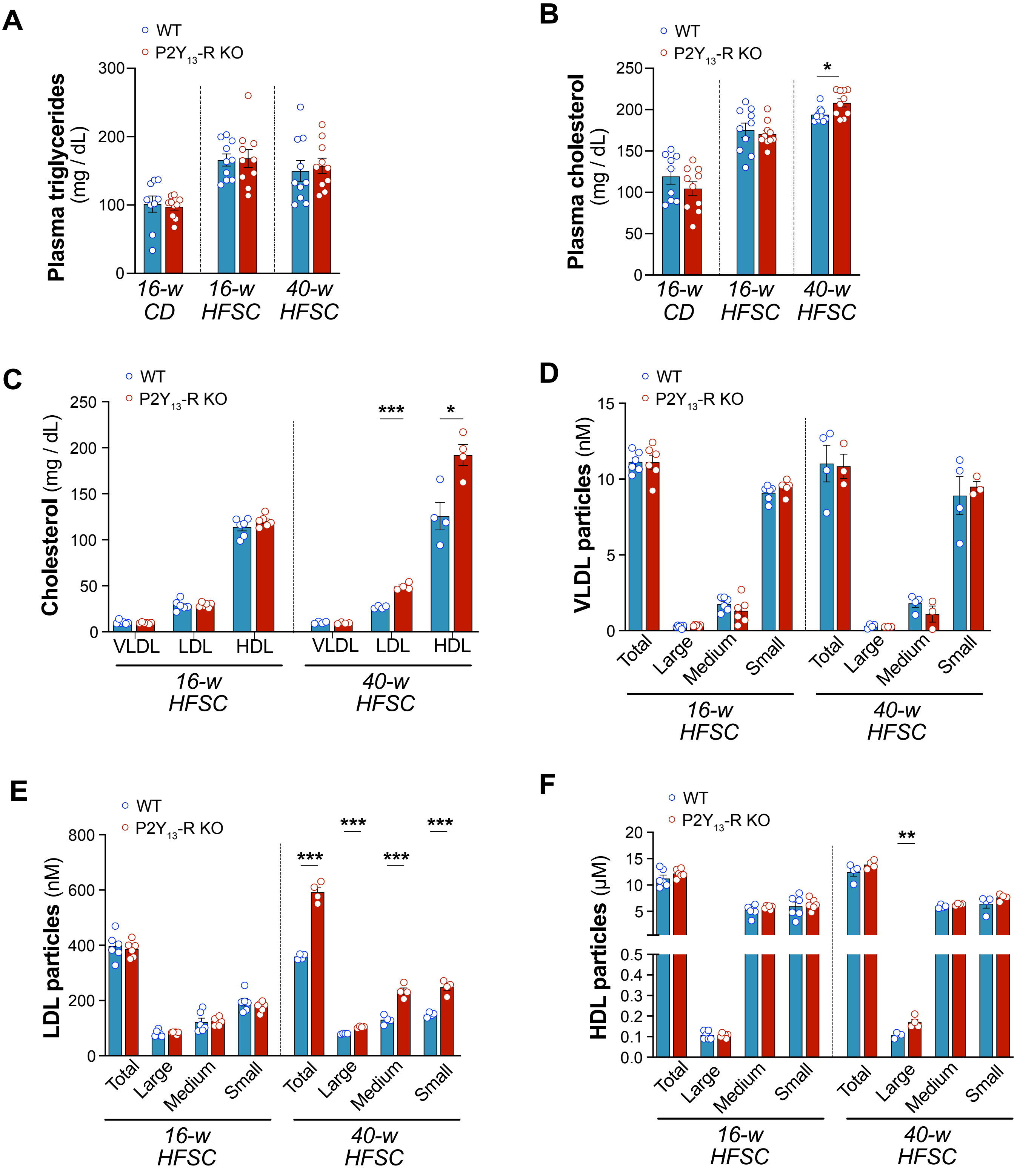

Supplemental Figure 3. Plasma lipids levels throughout experiments.

(A) Plasma concentrations of triglycerides (n = 9 or 10 mice per group).

(B) Plasma concentrations of total cholesterol (n = 9 or 10 mice per group).

(C) Plasma concentrations of cholesterol content in lipoprotein classes (n = 4 to 6 mice per group).

(D) Plasma concentrations of VLDL particles (n = 4 to 6 mice per group).

(E) Plasma concentrations of LDL particles (n = 4 to 6 mice per group).

(F) Plasma concentrations of HDL particle (n = 4 to 6 mice per group).

Open blue and red circles represent WT and $P2Y_{13}$-R KO mice, respectively.

All data are expressed as mean ± SEM.

*P<0.05, **P<0.01, ***P<0.001. (**A-F**, 2-tailed unpaired Student's t test was used for genotype comparison).

Results were obtained from mice fed chow diet for 16-weeks (16-w), or HFSC diet for 16- or 40-weeks (40-w).

CD, chow diet; HDL, high-density lipoprotein; HFSC, high-fat high-sucrose high-cholesterol; KO, knock-out; LDL, low-density lipoprotein; VLDL, very low-density lipoprotein; WT, wild-type.

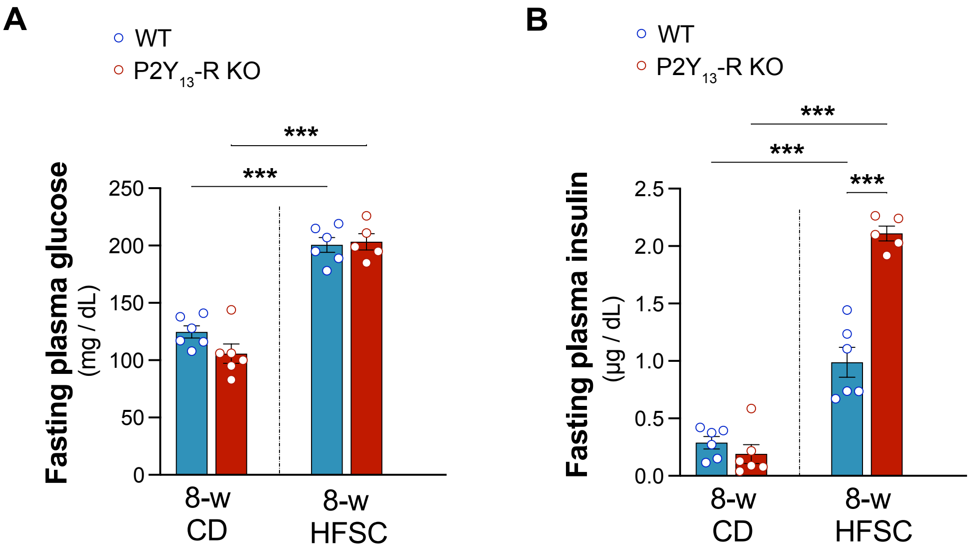

Supplemental Figure 4. HFSC feeding efficiently impairs glucose metabolism.

(A) Plasma levels of fasting glucose (n = 5 or 6 mice per group).

(B) Plasma level of fasting insulin (n = 5 or 6 mice per group).

Open blue and red circles represent WT and P2Y$_{13}$-R KO mice, respectively.

All data are expressed as mean ± SEM.

***P<0.001 (**A** and **B**, 1-way ANOVA followed by Holm-Sidak post hoc test was used for group comparison).

Results were obtained from mice fed CD or HFSC diet for 8-weeks (8-w)

CD, chow diet; HFSC, high-fat high-sucrose high-cholesterol.

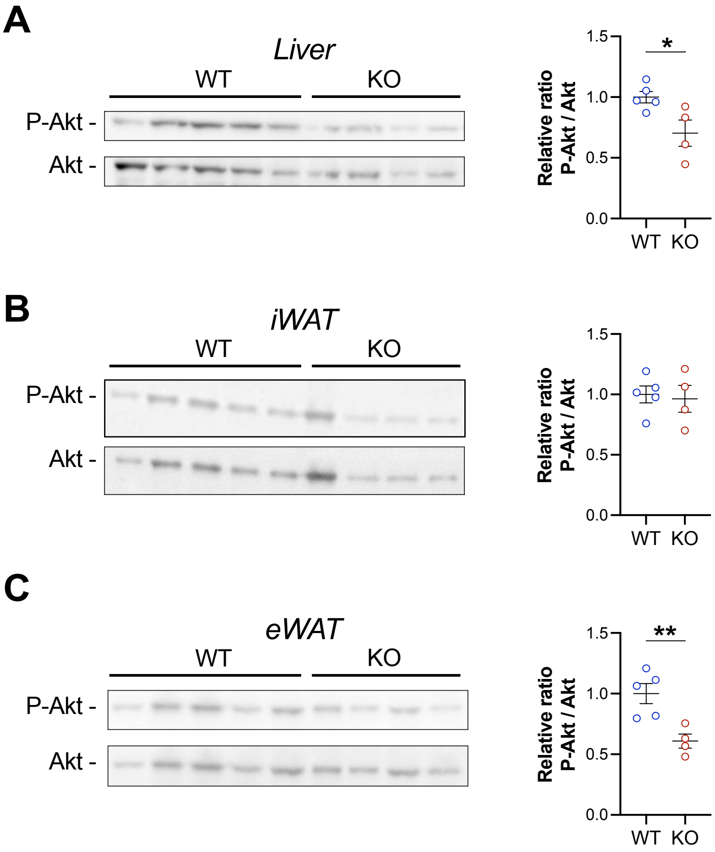

Supplemental Figure 5. Lack of $P2Y_{13}$-R decreases liver and white adipose tissue insulin sensitivity.

Western blot analysis and quantification of immunoblotting data of insulin sensitivity through Akt signaling in **(A)** liver (n = 4 or 5 mice per group), **(B**) iWAT (n = 4 or 5 mice per group), and **(C)** eWAT (n = 4 or 5 mice per group).

Open blue circles represent WT mice and open red circles represent $P2Y_{13}$-R KO mice.

All data are expressed as mean ± SEM.

*P<0.05, **P<0.01 (**A-F**, 2-tailed unpaired Student's t test was used for genotype comparison).

Results were obtained from mice fed HFSC diet for 10-weeks and received insulin at a dose of 1 mU / g of body weight into the portal vein.

eWAT, epididymal white adipose tissue; HFSC, high-fat high-sucrose high-cholesterol; iWAT, inguinal white adipose tissue; KO, knock-out; WT, wild-type.

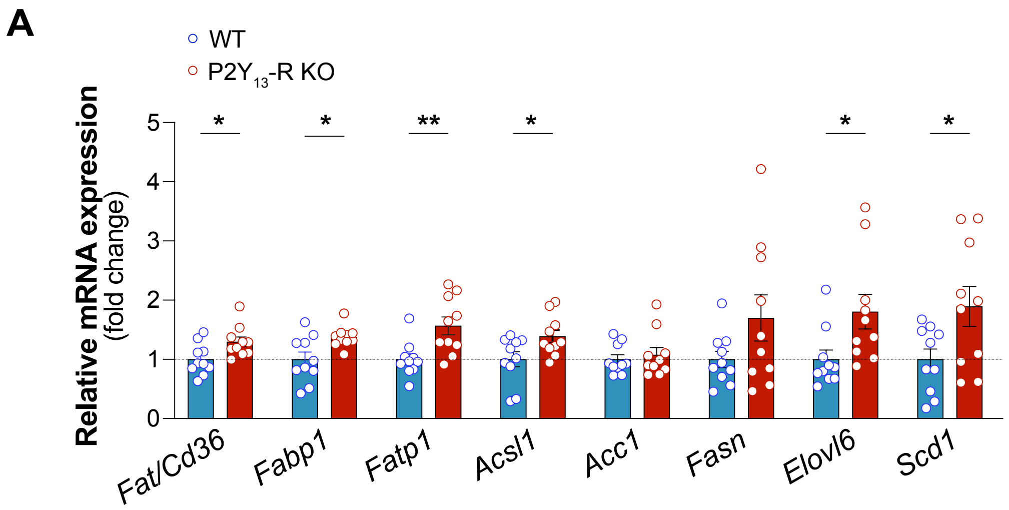

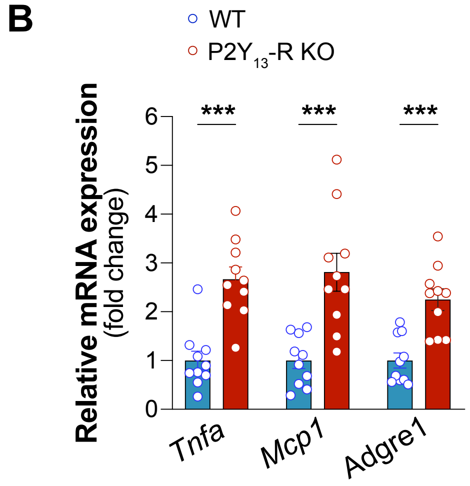

Supplemental Figure 6. Lack of P2Y$_{13}$-R increases hepatic expression of genes related to fatty acids handling, de-novo lipogenesis and inflammation. **(A)** Relative mRNA expression level of genes involved in fatty acids handling and de-novo lipogenesis in liver from WT and P2Y$_{13}$-R KO mice. **(B)** Relative mRNA expression level of genes related to inflammation in liver from WT and P2Y$_{13}$-R KO mice.

mRNA expression data were normalized relative to the expression of *Rps29*.

Open blue circles represent WT mice and open red circles represent P2Y$_{13}$-R KO mice (n = 10 mice per group).

All data are expressed as mean ± SEM. *P<0.05, **P<0.01 (2-tailed unpaired Student's t test was used for genotype comparison). All results were obtained from mice fed HFSC diet for 16-weeks.

Acc1, Acetyl-CoA carboxylase 1; *Acsl1*, Acyl-coA synthetase long chain family member 1;
Adgre1 (*F4/80*), adhesion G protein-coupled receptor E1*;* *Elovl6*, Elongation of long-chain fatty acids family member 6;
*Fabp*1, Fatty acid-binding protein 1; *Fasn*, Fatty acid synthase; *Fat/Cd36*, Fatty acid transporter;
Fatp1, Long-chain fatty acid transport protein 1; *Mcp1*, monocyte chemoattractant protein-1; *Rps29*, ribosomal protein S29;
Scd1, Stearoyl-CoA desaturase-1; *Tnfa,* Tumor necrosis factor alpha.

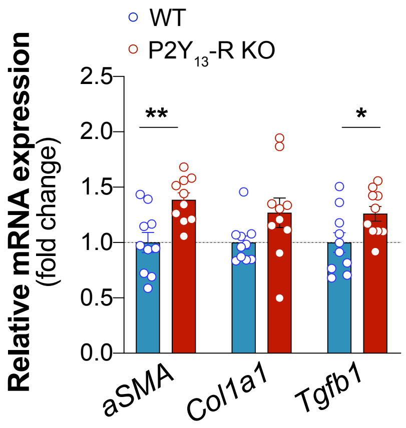

Supplemental Figure 7. Liver expression of genes involved in fibrosis.

Relative mRNA expression levels of fibrotic genes in liver from WT and $P2Y_{13}$-R KO mice.

mRNA expression data were normalized relative to the expression of *Rps29*.

Open blue circles represent WT mice and open red circles represent $P2Y_{13}$-R KO mice (n = 10 mice per group).

All data are expressed as mean ± SEM.

All results were obtained from mice fed HFSC diet for 40-weeks.

*P<0.05, **P<0.01 (2-tailed unpaired Student's t test was used for genotype comparison).

aSMA, alpha-smooth muscle actin; *Col1a1*, collagen type I alpha 1; *Rps29*, ribosomal protein S29

Tgfb1, transforming growth factor beta 1.

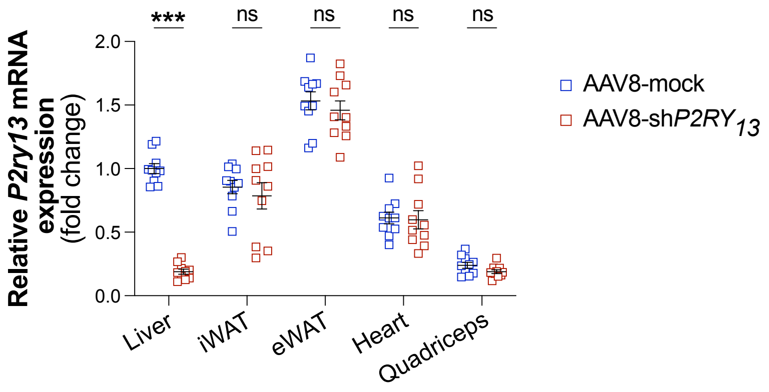

Supplemental Figure 8. P2Y$_{13}$-R expression in liver-specific P2Y$_{13}$-R KD mice.
The relative mRNA expression level of *p2ry13* was examined in tissues of mice transduced with AAV8 carrying either mock (AAV8-mock, represented by open blue square, n = 10) or shRNA targeted against P2Y$_{13}$-R (AAV8-sh*P2ry13*, represented by open red square, n = 10).
mRNA expression data were normalized relative to the expression of *Rps29*.
All data are expressed as mean ± SEM.
***P<0.001 (2-tailed unpaired Student's t test was used for group comparison).
Results were obtained from mice fed a HFSC diet for 16-weeks.
AAV8, adeno-associated virus serotype 8; eWAT, epididymal white adipose tissue;
HFSC, high-fat high-sucrose high-cholesterol; iWAT, inguinal white adipose tissue;
KD, knock-down; ns, non-significant.

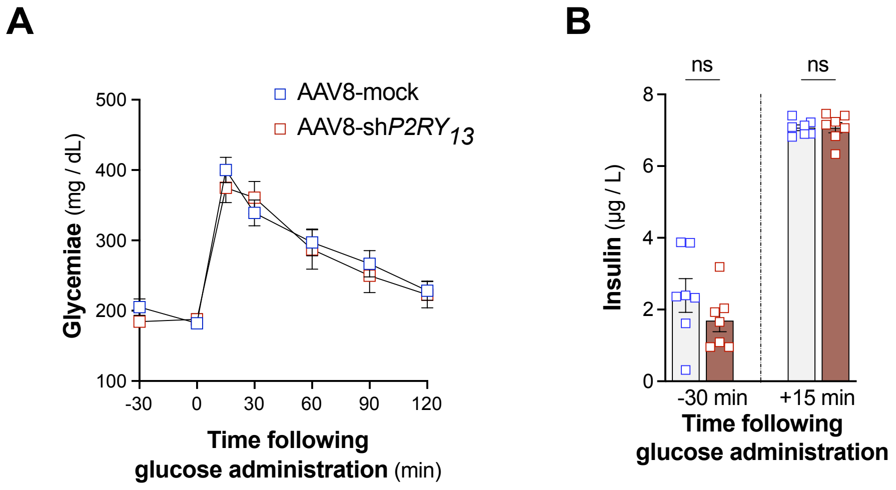

Supplemental Figure 9. Glucose metabolism in liver specific P2Y_{13}-R KD mice fed a HFSC diet.

(A) OGTT (3 g/kg glucose) (n = 9 or 10 mice per group)

(B) OGTT-associated basal and glucose-stimulated insulinemia values in overnight-fasted (n = 7 mice per group)

Open blue squares represent control mice, transduced with AAV8-mock, and

open red squares represent P2Y_{13}-R KD mice, transduced with AAV8-sh*P2RY13*.

All data are expressed as mean ± SEM (**A**, 2-way ANOVA followed by Bonferroni's post hoc test was used for group comparison; **B**, 2-tailed unpaired Student's t test was used for group comparison).

All results were obtained from mice fed a HFSC diet for 8-weeks.

AAV8, adeno-associated virus serotype 8; HFSC, high-fat high-sucrose high-cholesterol; KD, knock-down; ns, non-significant; OGTT, oral oral glucose tolerance test.

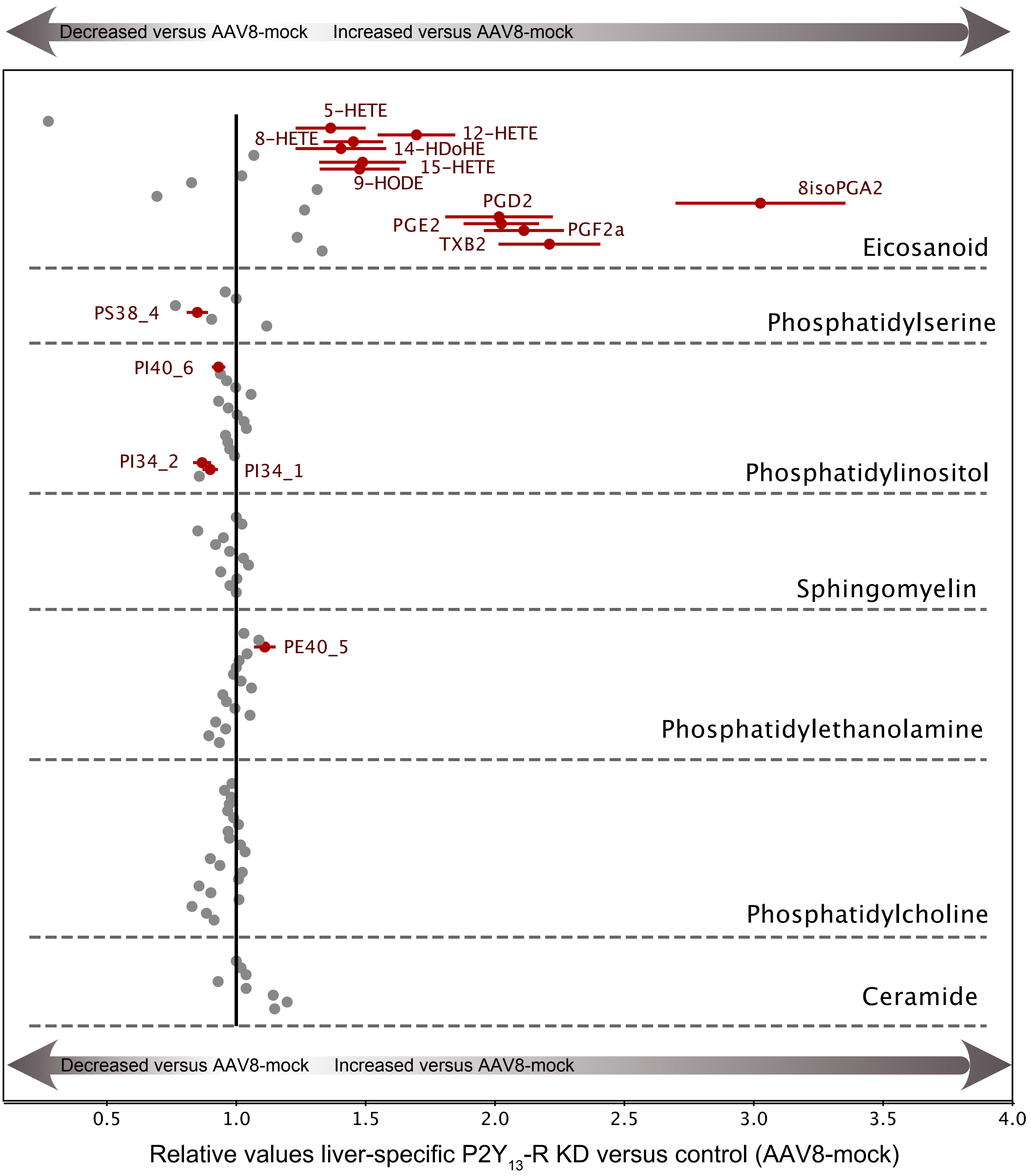

Supplemental Figure 10. Liver lipidome analysis in liver-specific $P2Y_{13}$-R KD fed HFSC for 16-weeks

A comparative liver lipidome analysis between $P2Y_{13}$-R KD (AAV8-sh*P2RY*$_{13}$) and control (AAV8-mock) mice was conducted regarding phospholipids and eicosanoids after 16 weeks of HFSC diet feeding.

Data are presented as the mean of the $P2Y_{13}$-R KD relative values to control ± SEM.

Results were obtained from 10 mice per groups.

Significant lipid changes ($p<0.05$) are identified and highlighted in red.

HFSC, high-fat high-sucrose high-cholesterol; KD, knock-down.
