# Supplementary figures and images for: P2Y13 receptor deficiency favors adipose tissue lipolysis and worsens insulin resistance and fatty liver disease
Source: JCI Insight. 2024 Mar 12;9(8):e175623. doi: 10.1172/jci.insight.175623 (PMC11141875; doi:10.1172/jci.insight.175623)

Full unedited gel for Figure 5D

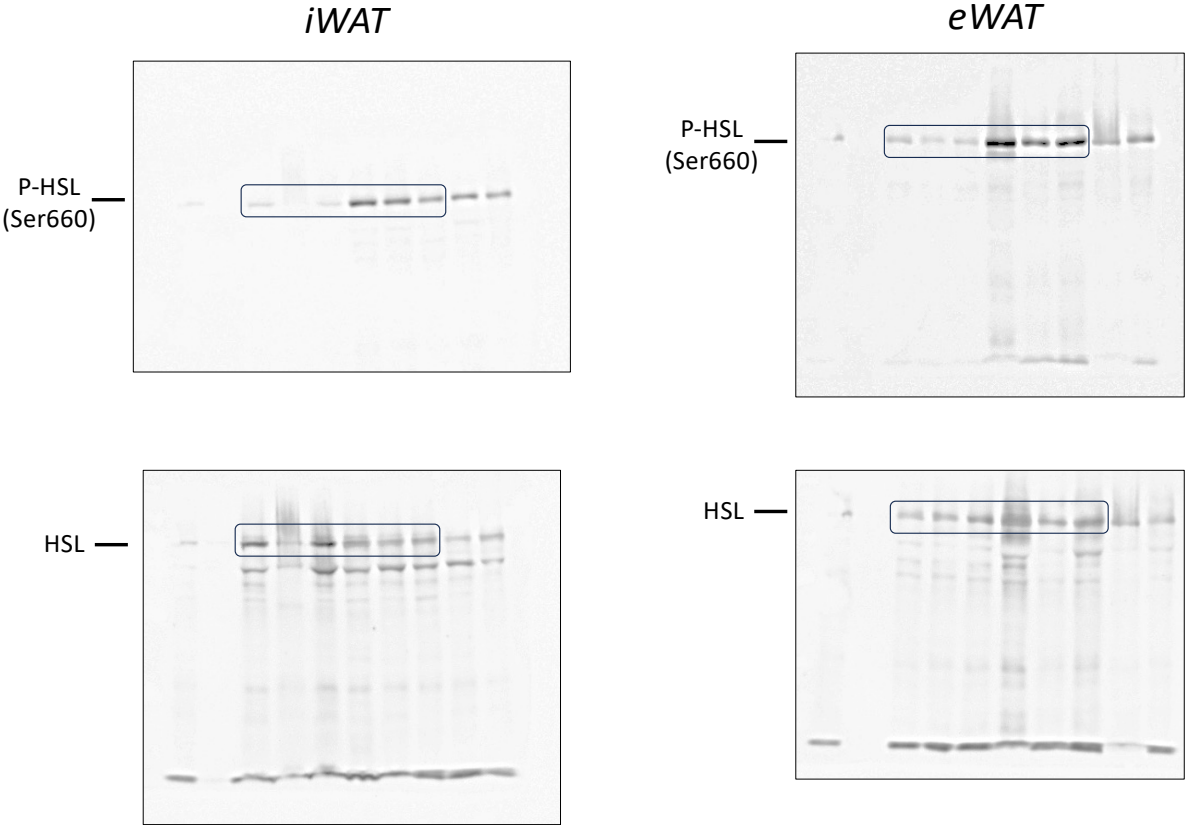

Full unedited gel Supplemental Figure 5

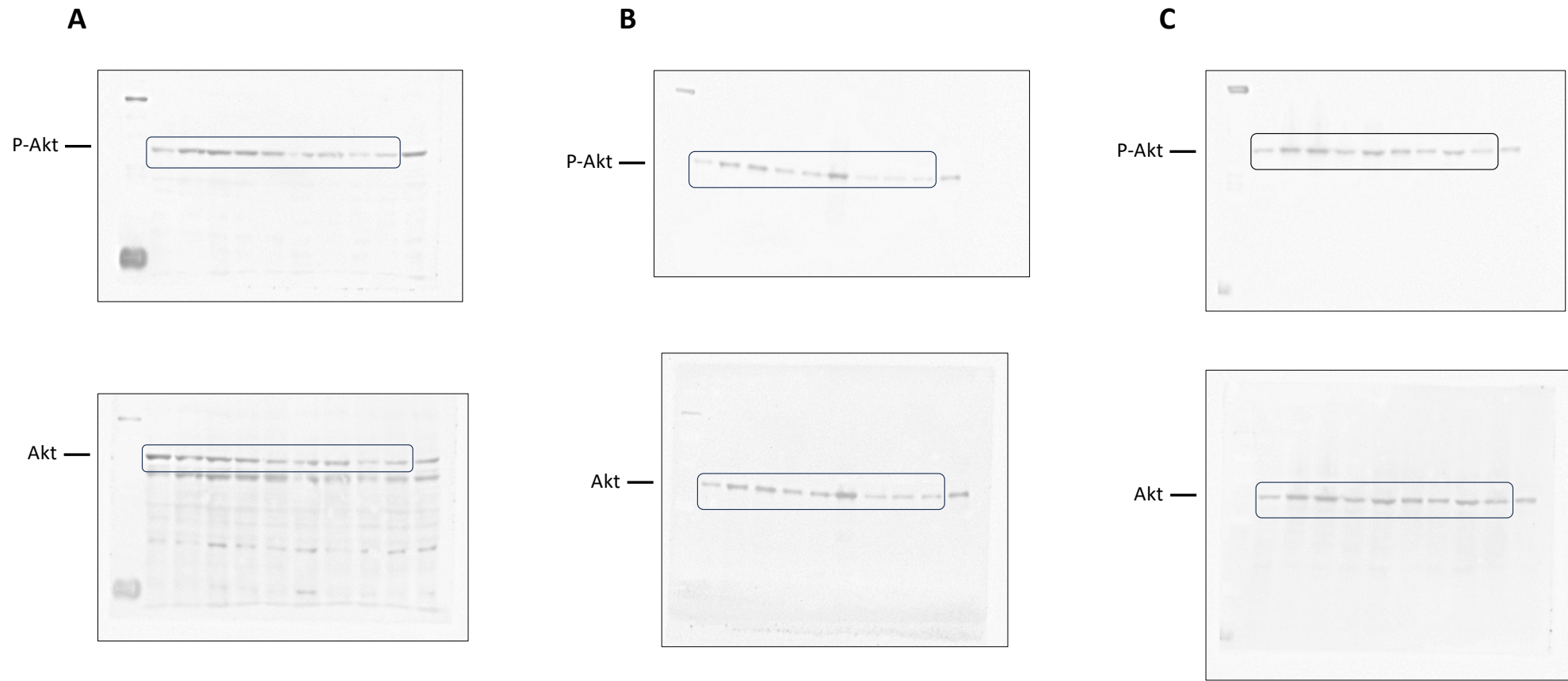

Supplement: Unedited blot and gel images [file jciinsight-9-175623-s163.pdf]
